# Neural Stem Cell Gene Therapy Ameliorates Pathology and Function in a Mouse Model of Globoid Cell Leukodystrophy

**DOI:** 10.1002/stem.701

**Published:** 2011-08-01

**Authors:** Margherita Neri, Alessandra Ricca, Ilaria di Girolamo, Beatriz Alcala'-Franco, Chiara Cavazzin, Aldo Orlacchio, Sabata Martino, Luigi Naldini, Angela Gritti

**Affiliations:** a Division of Regenerative Medicine, Stem Cells and Gene Therapy, San Raffaele Scientific Institute, Telethon Institute for Gene Therapy (HSR-TIGET)Milano, Italy; b Vita-Salute San Raffaele UniversityMilano, Italy; c Department of Experimental Medicine and Biochemical Sciences, Section of Biochemistry and Molecular Biology, University of PerugiaPerugia, Italy

**Keywords:** Lysosomal storage diseases, Leukodystrophies, Nervous system, Neural stem cells, Gene therapy, Stem cell transplantation

## Abstract

Murine neural stem cells (mNSCs), either naive or genetically modified to express supranormal levels of β-galactocerebrosidase (GALC), were transplanted into the brain of Twitcher mice, a murine model of globoid cell leukodystrophy, a severe sphingolipidosis. Cells engrafted long-term into the host cytoarchitecture, producing functional GALC. Levels of enzyme activity in brain and spinal cord tissues were enhanced when GALC-overexpressing NSC were used. Enzymatic correction correlated with reduced tissue storage, decreased activation of astroglia and microglia, delayed onset of symptoms, and longer lifespan. Mechanisms underlying the therapeutic effect of mNSC included widespread enzyme distribution, cross-correction of host cells, anti-inflammatory activity, and neuroprotection. Similar cell engraftment and metabolic correction were reproduced using human NSC. Thus, NSC gene therapy rapidly reconstitutes sustained and long-lasting enzyme activity in central nervous system tissues. Combining this approach with treatments targeting the systemic disease associated with leukodystrophies may provide significant therapeutic benefit. Stem Cells 2011;29:1559–1571

## INTRODUCTION

Globoid cell leukodystrophy (GLD or Krabbe disease) is a monogenic lysosomal storage disorder (LSD) [1] caused by the deficiency of β-galactocerebrosidase protein (GALC) [2]. The abnormal accumulation of glycosphingolipids within lysosomes is considered to be the critical pathogenetic mechanism leading to oligodendrocyte and Schwann cell death throughout the central nervous system (CNS) and peripheral nervous system (PNS) [2, 3]. The widespread demyelination and neurodegeneration are worsened by extensive neuroinflammation, a hallmark that characterizes these and other LSD [1]. The most common phenotype of GLD is the rapidly progressive early infantile form, which manifests within the first 6 months of life and is characterized by severe neuro-developmental delay, progressive motor dysfunction, and early death [4].

A severe hurdle to the treatment of GLD is the inability to circumvent the blood brain barrier to deliver effective levels of therapeutic enzyme to CNS tissues. Hematopoietic cell transplant (HCT) using bone marrow and umbilical cord blood cells from healthy donors have the potential to deliver functional enzyme to the CNS and PNS by macrophage/microglia replacement with donor-derived cells [5]. The outcome of these approaches in LSD patients mainly depends on the rapidity of progression, on the level of CNS involvement characterizing the diseases themselves and on the extent of enzymatic reconstitution achieved in relevant target tissues [6, 7]. Little is known regarding the kinetics post-transplant of microglia reconstitution in the human CNS. In mice this process might require up to 6 months, according to the myeloablative regimen used and the underlying disease [8]. A slow microglial turnover might preclude obtaining sufficient levels of corrective enzyme early in postnatal CNS development, which is critical for myelination and for the correct development of CNS connections. This might account for the lack of definitive treatment by conventional HCT approach in infantile and early juvenil Krabbe patients [6, 9, 10], in which white matter tracts are severely compromised already at birth [11]. In this perspective, approaches ensuring fast, sustained and widespread enzymatic activity in CNS tissues before the onset of symptoms or, at least, at the very early stages of the diseases, might be of relevance to counteract/prevent irreversible CNS damage.

Direct intracerebral injection of vectors [12] or transplantation of enzyme-producing cells [13] might achieve this goal. In the latter approach, the therapeutic plasticity of neural stem cells (NSC) might be exploited [14, 15]. NSC [16–18] promote donor-to-recipient cross correction via enzyme secretion-recapture [19], but can also mediate cell replacement [20], cell rescue through trophic support [21–23] and other mechanisms such as immunomodulation [24, 25] and neuroprotection [21, 26, 27]. Thus, the peculiar NSC biology, coupled to the secretion of functional GALC may have an impact on the global neuropathology that characterizes GLD and similar neurodegenerative LSD [21, 27–30].

Previous studies have tested NSC-based approaches in the Twitcher mouse, using murine NSC (mNSC) and human NSC (hNSC) [31–33], an immortalized NSC clone [32, 33] or a transformed astrocytic progenitor cell line [34]. Overall, these studies indicated that NSC/progenitor cell types engraft in the Twitcher brain and secrete the GALC enzyme, providing therapeutic benefit, although no definitive cure. However, important issues such as the long-term survival of NSC in the toxic Twitcher environment, the efficacy of NSC transplants in counteracting tissue damage and inflammation in widespread CNS regions and the potential advantage of enzyme overexpression in donor cells [31, 33] to achieve therapeutic benefit, still remained controversial. In addition, the use of transformed cell lines, which in one case resulted in the formation of brain tumors [34] undermined the relevance of some of these studies.

The goal of our study was to assess the therapeutic potential of an ex vivo NSC gene therapy approach in Twi mice. We comprehensively modeled the engraftment, migration, and differentiation of somatic NSC after neonatal intracerebroventricular transplantation. We first used syngenic mNSC isolated and cultured from the postnatal brain subventricular zone (SVZ) [17, 35], either naive or genetically modified to express supraphysiological levels of GALC. NSC rapidly engrafted, migrated and stably expressed the enzyme, which was widely distributed through the cerebrospinal fluid (CSF) flow to reach the whole brain and the spinal cord (SC). Supraphysiological levels of GALC in donor cells resulted in enhanced metabolic reconstitution of host CNS tissue, which was mediated by cross-correction, the mechanism by which secreted lysosomal enzymes are uptaken by neighboring cells. Enzymatic correction correlated with reduction of tissue storage, decrease of activated astroglia and microglia, modulation of inflammation, and improved survival. These promising therapeutic benefits were reproduced using hNSC, which showed similar pattern of cell engraftment, distribution, and metabolic reconstitution.

## MATERIALS AND METHODS

### Mouse Strains

Twitcher mice bear a spontaneous point mutation resulting in a premature stop codon and no residual GALC activity [36]. FVB/Twi mice were generated in our animal research facility by breeding heterozygous Twi (+/−) C57BL6 mice (Jacson Laboratories, Bar Harbor, Maine) with wild-type (WT) (+/+) Friend leukemia virus B (FVB) mice. FVB/Twicher mice (from hereon called Twi mice) show a slower progressive form of the disease than the canonical Twicher mice and have more numerous litters. Tremors develop at around postnatal day 21 (PND21), and progress to severe resting tremor, weight loss, paralysis and wasting of hind legs. At 40 days of age, the PNS is severely demyelinated, while CNS tissues show a milder and patchy demyelination. Death occurs within 45–50 days of age. Age-matched Twi+/+ littermates were used as wild-type controls for Twi−/− mice in all the experimental conditions.

Metachromatic leukodystrophy (MLD) mice used to isolate mNSC were described previously [19]. Mouse colonies were maintained in the animal facility of the Fondazione San Raffaele del Monte Tabor, Milano, Italy. Procedures were performed according to the protocols approved by the Animal Care and Use Committee of the Fondazione San Raffaele del Monte Tabor (IACUC #314 and #420).

### Vector Production

The bidirectional (bd) lentiviral vectors (LV) used in this study were used previously and described [19, 37]. The control (CTRL) vector encodes for green fluorescent protein (GFP) and the truncated form of the low affinity nerve growth factor receptor (bdLV.ΔNGFR.GFP, referred to as bdLV.CTRL); the therapeutic vector encodes for green fluorescent protein and for the murine GALC cDNA C-terminally tagged with the human influenza hemagglutinin epitope (HA) (bdLV.GALC-HA.GFP, referred to as bdLV.GALC). Monocistronic LV encoding for the human arylsulfatase A (ARSA) cDNA were described previously [8, 38]. Reagents, detailed cloning procedures and sequence information are available upon request. See Supporting Information Experimental Procedures for details on LV preparation.

### NSC Cultures and LV Transduction

Murine NSC and hNSC cultures were established and expanded in serum-free medium (Dulbecco's modified Eagle's medium/F12 1:1 vol:vol containing insulin, apo-transferrin, putrescine, and progesterone) containing fibroblast growth factor-2 (FGF2) and epidermal growth factor (EGF) (10 and 20 ng/ml, respectively; Peprotech, Rocky Hill, NY) (growth medium), according to the neurosphere assay [17]. Long-term proliferation, self-renewal, and multipotency were routinely assessed by functional assays [35, 39]. LV transduction was performed on serially subcultured NSC (passages 5–10 and passages 18–25 for mNSC and hNSC, respectively). See Supporting Information Experimental Procedures for details on NSC isolation, propagation, and transduction.

### Cross-Correction Experiments

WT untransduced (UT) or gene-corrected Twi mNSC were differentiated and used as the source of secreted enzyme (donor cells), as described previously [19]. UT-Twi and MLD NSC cultures (acceptor cells), at the same stage of differentiation as donor cells, were exposed to the supernatant conditioned by donor cells in the presence or absence of mannose-6-phosphate (M6P; 5 mM) for the last 72 hours of culture. After a 24-hour washout with fresh medium, donor cells, acceptor cells, and their supernatants were collected and analyzed for enzyme activity.

### Transplantation

LV-transduced neurosphere bulk cultures (3–10 subculturing passages post-transduction, corresponding to 15–40 days in culture) were enzimatically dissociated (Accumax, Millipore, Temecula, CA, 3 minutes at 37°C) and plated in growth medium. Cells were collected 48 hours later, centrifuged, mildly mechanically dissociated, counted by Trypan Blue exclusion (viability >90%), and resuspended (2.5 × 10^5^ cell/μl) in PBS + 0.1% DNase (Sigma, St. Louis, MO). Cells were kept in ice and used within 1 hour. Cell viability assessed at the end of the transplantation procedure was >80%. Newborn mice (PND2) were used as recipients. Animal heads were trans-illuminated to identify the lateral ventricles and NSC suspensions were rapidly injected (1 or 2 μl/injection site; bilateral) through trans-cutaneous insertion of the tip of a hand-drawn glass capillary without exposing the skull. Coordinates for ventricular injections were determinated in pilot dye injection experiments. The procedure takes less than 5 minutes for each mouse, then neonates are immediately returned to parental cures. The overall survival of injected mice was around 90%.

We transplanted a total of *n* = 110 mice (104 mice with mNSCs and six mice with hNSCs). A group of untreated Twi (*n* = 62) and WT littermates (*n* = 23) were included as controls. Details on the experimental groups are available in Supporting Information Table 2.

### Tissue Processing

Animals were euthanized at PND7 and PND40. A group of transplanted animals was allowed to live until terminal stage (body weight <80% than age-matched WT mice or inability to eat and drink) to monitor survival. Tissues to be used for GALC activity measurment and for mRNA profiling were collected following perfusion of animals with saline, to remove contaminating blood. Brains were cut in two hemispheres and the telencephalon was separated from the cerebellum and pons. SC was collected as a whole. Tissues were either quickly frozen in liquid nitrogen or immediately processed to obtain tissue extracts. Cerebrospinal fluid (CSF) was collected from the cysterna magna immediately prior to euthanasia using a hand-drawn glass capillary.

A group of mice were intracardially perfused via the descending aorta (under deep anesthesia) with 0.9% NaCl followed by 4% paraformaldheyde (PFA) in phosphate buffer solution (PBS). Brains and SC tissues were collected and equilibrated for 24 hours in 4% PFA in PBS, washed in PBS+NaN_3_, and then included in 4% agarose. Serial coronal vibratome-cut sections (six series, 40 μm thick) were processed for histology and immunofluorescence analyses.

### Immunohistochemistry, Immunofluorescence, and Histopathology

Immunofluorescence and histochemistry were performed on free-floating vibratome sections as described previously [19]. See Supporting Information Experimental Procedures for detailed protocols and Supporting Information Table 1 for the list of antibodies used.

### Cell Counts and Image Acquisition

The number of engrafted cells was assessed in 40 μm thick coronal brain sections (15–18 sections per mice, corresponding to one out of six series) using anti-green fluorescence (GFP) antibody, and expressed as the number of cells per section. Details on cell counts and image acquisition have been described previously [19] and are summarized in Supporting Information Experimental Procedures.

### Determination of Enzyme Activity

GALC and arylsulfatase A (ARSA) activity were measured according to previously described assays [40–42]. See Supporting Information Experimental Procedures for detailed protocols regarding the preparation of brain and cell extracts. S300 gel filtration chromatography was performed as described previously [19].

### Gait Analyses

Walking ability of PND40 mice was measured by applying food coloring to paws and allowing mice to walk on graph papers. Footprints from individual mice were collected and photographed.

### Statistics

Cell counts, enzyme activity, vector copy number (VCN) values, and data obtained following the quantification of immunopositive area by the ImageJ software were analyzed with Graph Pad Prism version 5.0a for Macintosh and expressed as the mean ± SEM. Unpaired Student's *t* test, Mann-Whitney test, one- or two-way analysis of variance (ANOVA) followed by Bonferroni, or Dunnett's posttests were used when appropriate (statistical significance: *p* < .05). Log-rank test was used to compare Kaplan-Meyer survival curves. The number of samples/mice and the statistical test used are indicated in the legends to each figure.

## RESULTS

### Expression, Secretion, and Recapture of the GALC Enzyme in mNSC Cultures

**Figure 1:**
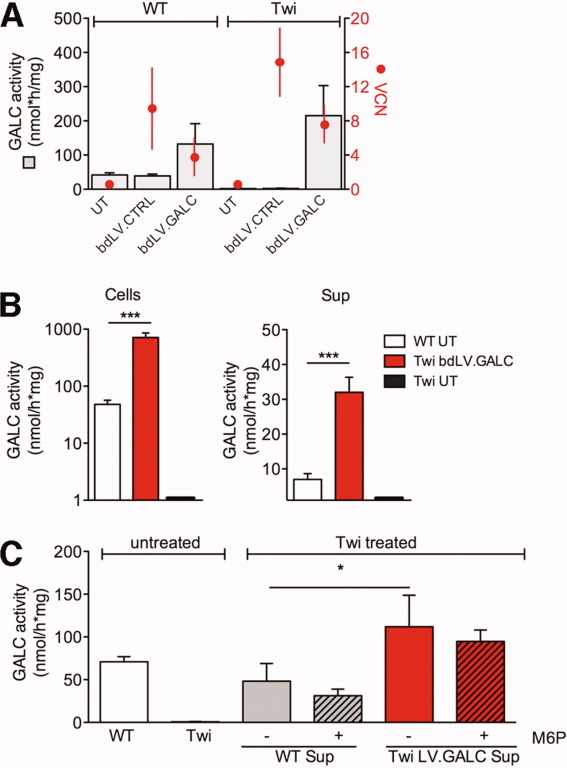
Wild-type (WT) and transgenic β-galactocerebrosidase (GALC) proteins are internalized by enzyme-deficient murine neural stem cell (mNSC) progeny. **(A):** Copy number of integrated lentiviral vector (LV) genome (vector copy number, VCN) and enzyme activity of wild-type (WT) and GALC-deficient Twi mice mNSC populations, either untransduced (UT) or transduced using bdLV.GALC and bdLV.CTRL vectors (3 × 10^7^ transducing units/ml) (*n* = 7 independent experiments). **(B):** Enzyme activity in cells and in supernatant (Sup) of GALC-overexpressing mNSC-derived differentiated progeny with respect to untransduced (UT) WT cells. **(C):** Intracellular GALC activity in Twi mNSC progeny (Twi treated cells) after cross-correction with supernatants of WT mNSC or of enzyme-overexpressing cells, in the presence or absence of free M6P. Data analyzed by Student's *t* test **(B)** (*n* = 5 independent experiments) and one-way analysis of variance (Kruskal-Wallis *H* test followed by Dunnett's multiple comparison test) **(C)** (*n* = 3 independent experiments). *, *p* < .05; ***, *p* < .001.

We transduced WT and GALC-deficient mNSC with a bidirectional lentiviral vector (bdLV) expressing the murine GALC and the reporter protein GFP (bdLV.GALC) applying previously optimized conditions [19]. This resulted in high transduction efficiency (70%–90% of GFP+ cells in the neurosphere bulk culture assessed by fluorescence activated cell sorting (FACS) analysis; vector copy number-VCN- ranging from 3 to 15), lysosomal localization of the transgenic GALC (Supporting Information Fig. 1A) and supranormal enzyme activity (two- to fivefold the WT levels) (Fig. 1A). Cell proliferation and multipotency of GALC-transduced (t) mNSC (GALCtNSC) were not impaired as compared with bdLV.CTRL-transduced and UT WT cells (CTRLtWT and UT WTNSC) (Supporting Information Fig. 1B–1D). The percentage of GFP+ cells in differentiated cultures was the same as in the neurosphere bulk cultures (70%–90%) and was composed by: ≅8% neurons (TUJ1+), ≅12% oligodendrocytes (GalCer+) and ≅80% astroglial (GFAP+) cells. These percentages were similar to those measured in differentiated cultures of untreated cells [43]. We observed a two to threefold increase in enzyme activity in the differentiated progeny of WT and enzyme-overexpressing mNSC (Supporting Information Fig. 1E).

Higher enzyme activity in gene-corrected mNSC correlated to higher enzyme activity in the supernatant (Fig. 1B), likely due to increased enzyme secretion. Lysosomal enzymes can be secreted in the extracellular milieau and can be recaptured by neighboring cells mainly through the mannose-6-phosphate (M6P) receptor (cross-correction) [44]. Exposure of enzyme-deficient mNSC progeny (acceptor cells) to the supernatant of enzyme-overexpressing cells resulted in more efficient cross-correction, as shown by higher intracellular GALC activity measured in acceptor cells (Fig. 1C). Addition of the competitive inhibitor mannose-6-phosphate (M6P), which markedly inhibited ARSA reuptake in ARSA-deficient mNSC cultures in similar experimental conditions (Supporting Information Fig. 2) slightly affected GALC uptake in GALC-deficient mNSC progeny (Fig. 1C). These results indicate that gene transfer corrects GALC deficiency of Twi mNSC and enhances the extent of cross-correction of nontransduced cells. They also suggest the occurrence of M6P receptor(R)-independent reuptake of GALC in mNSC-derived progeny. We expected that mNSC may similarly cross-correct enzyme-deficient host cells following transplantation in the CNS of GALC-deficient mice.

### Engraftment and Distribution of mNSC in the Twi Brain

We evaluated whether mNSC engraft, survive, migrate and differentiate following transplantation in GALC-deficient Twi mice, also addressing a potential advantage of enzyme-overexpressing mNSC on their WT counterpart. Asymptomatic Twi neonates (PND2) and age-matched WT littermates were transplanted in the cerebral ventricles with Twi or WT mNSC, transduced with either bdLV.CTRL (CTRLtTwiNSC and CTRLtWTNSC, expressing no activity and physiological GALC activity, respectively) or bdLV.GALC (GALCtTwiNSC and GALCtWTNSC, expressing supranormal GALC activity). Details regarding experimental groups are available in Supporting Information Table 2. We used two different CTRLtWTNSC lines (GALC activity: 30–50 nmol/h/mg) and five different GALCtNSC lines (three Twi NSC and 2 WT NSC, four independent transduction experiments; GALC activity: 200–300 nmol/h/mg).

**Figure 2:**
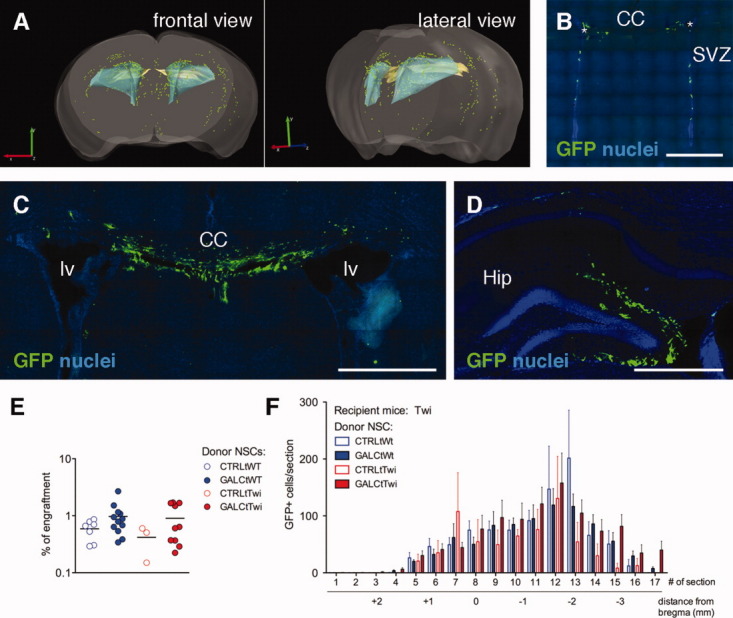
Engraftment and distribution of murine neural stem cell (mNSC) transplanted into the Twi brain. **(A):** Three-dimensional reconstruction of a representative Twi mouse brain transplanted with CTRLtWTNSC. Forebrain, gray; lateral ventricles, light blue; white matter, light yellow; engrafted GFP+ cells, green dots. **(B–D):** GFP+ cells in the forebrain subventricular zone lining the lateral ventricles (asterisks in B), in the corpus callosum **(C)** and in the hippocampus **(D)**. Scale bars = 0.5 mm **(B–D)**. **(E):** Percentage of engraftment in Twi mice injected with wild-type and Twi mNSC transduced with bdLV.CTRL or bdLV.GALC. Data analyzed by one-way ANOVA (Kruskal-Wallis *H* test followed by Dunnett's multiple comparison test), *p* = .2. **(F):** Rostrocaudal distribution of engrafted GFP+ cells (experimental groups as described in **E**). *n* = 3–12 mice per treatment group. Data analyzed by two-way analysis of variance, *p* = .1245. Abbreviations: CC, corpus callosum; GFP, green fluorescence protein; Hip, hippocampus; lv, lateral ventricle; SVZ, subventricular zone.

Mice were examined at PND7 and PND40 to evaluate the engraftment and fate of transplanted cells, which were detected using an anti-GFP antibody. Indirect immunofluroscence (IF) assay on serial rostrocaudal coronal brain sections from PND40 brains followed by confocal analysis and three-dimensional reconstruction (Fig. 2A) showed the presence of GFP+ cells throughout the forebrain, distributed in the parenchyma as well as lining the lateral ventricles (Fig. 2B), with a preference for subcortical white matter tracts (Fig. 2C; corpus callosum) and the hippocampus (Fig. 2D). We found comparable numbers (Fig. 2E) and similar distribution (Fig. 2F) of engrafted cells in Twi mice transplanted with the different mNSC preparations. The yield of engraftment ranged between 0.15% and 2.6% (the percentages might be slightly underestimated since we transplanted bulk NSC preparations containing 80%–90% of GFP+ cells). Engrafted cells were found in a region comprised within 2.0 mm rostral and 3.0 mm caudal from bregma. Few if any cells were found in the cerebellum, brainstem, and SC. This pattern of cell engraftment and distribution was similar to that found in PND40 WT mice transplanted as newborns with GALCtWTNSC, suggesting that NSC integrate and survive in the inflammatory Twi environment (Supporting Information Fig. 3A). Importantly, GFP+ cells were found in transplanted Twi mice as early as 5 days after transplant (PND7; Supporting Information Fig. 3B), with a yield of engraftment comparable with that measured in Twi brains at PND40 and at terminal stage (Supporting Information Fig. 3C). A similar yield of engraftment was measured in brain tissues of PND90 WT mice transplanted at birth with GALCtWTNSC (Supporting Information Fig. 3C), which were included as controls to check the long-term survival of engrafted NSC. Finally, we found similar numbers of engrafted cells in the brains of Twi mice injected with either cell dose of 0.5 × 10^6^ (5,208 ± 871 cells; *n* = 18) and 1 × 10^6^ (5,540 ± 799 cells; *n* = 15), suggesting that the majority of injected cells are cleared in the CSF and that the egress from the ventricles is the limiting step for proficient engraftment in the brain parenchyma.

**Figure 3:**
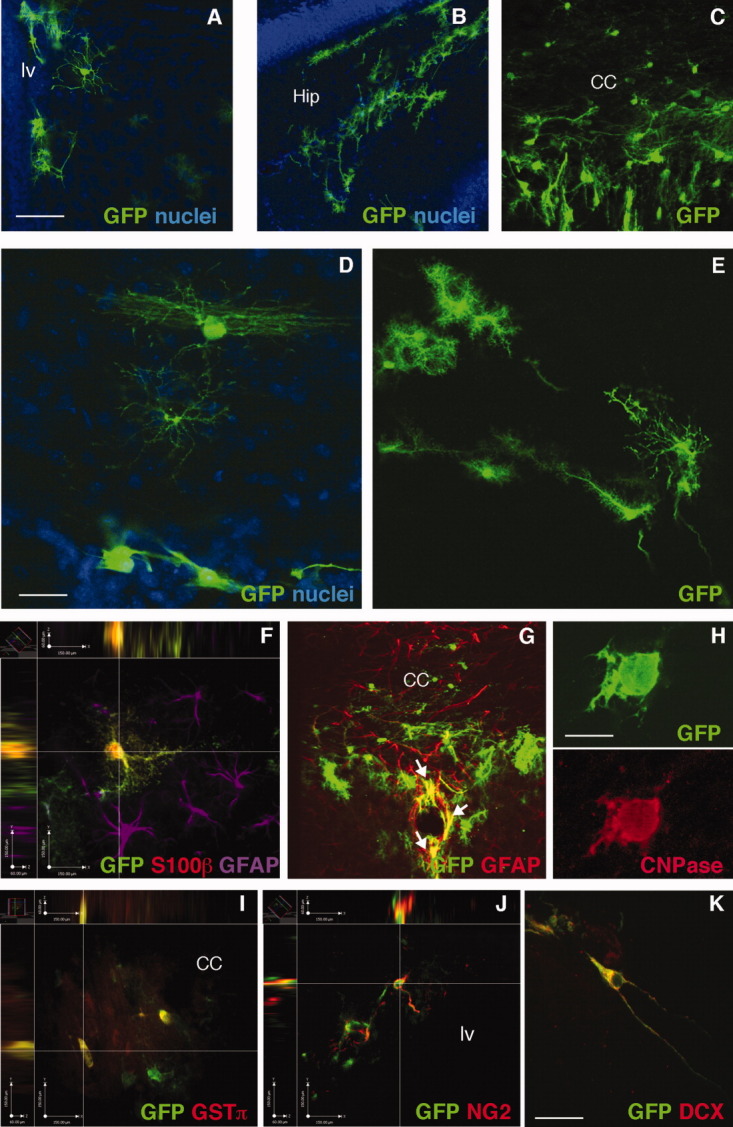
Morphology and lineage specification of murine neural stem cells transplanted within the Twi central nervous system. **(A–C):** Representative confocal images showing variable morphology of GALCtTwiNSC green fluorescence protein (GFP+), engrafted in the forebrain subventricular zone (SVZ) lining the lateral ventricles **(A)**, in the hippocampus **(B)**, and within or close to white matter tracts (i.e., corpus callosum [CC]; **C**). **(D, E):** GFP+ cells with oligodendroglial **(D)** and astroglial **(E)** morphology in white matter tracts. **(F–K):** Representative confocal merged pictures showing GFP+ cells expressing the astroglial markers S100β **(F**; CC) and glial fibrillary acidic protein (GFAP) **(G)**, the oligodendroglial markers CNPase (**H**; CC), GST-π **(I)**, NG2 (**J**; SVZ), and the neuronal progenitor marker doublecortin (**K**; hippocampal region). Yellow color represents merged signal. Double-labeled cells are indicated by arrows in **(G)**. Confocal *z*-stack pictures are shown in **(F)**, **(I)**, and **(J)**. Scale bars = 250 μm (**A–C**, shown in **A**), 25 μm (**D, E**, shown in **D**), 60 μm (**G, K**, shown in **K**), 10 μm **(H)**. Abbreviations: CNPase, 2′,3′-cyclic nucleotide 3′-phosphodiesterase; DCX, doublecortin; GFP, green fluorescence protein; GST, glutathione-S-transferase; Hip, hippocampus; lv, lateral ventricle; NG2, chondroitin sulfate proteoglycan.

We found expression of the proliferation marker Ki67 and of the apoptotic marker Cleaved Caspase three in <1% of GFP+ cells at all the time points analyzed (data not shown). This led us to exclude massive cell loss following cell engraftment and suggested initiation of lineage commitment. Cells engrafted in the periventricular region showed immature morphology (Fig. 3A; lateral ventricle, lv) while the majority of the cells engrafted in the hippocampus (Hip; Fig. 3B) and in white matter tracts (corpus callosum, CC; Fig. 3C–3E) were morphologically similar to astroglial and oligodendroglial cells. We assessed the fate of engrafted NSC in the Twi microenvironment by indirect IF using antibodies to GFP and to lineage-specific markers followed by confocal analysis. We found variable proportions of engrafted GFP+ cells espressing markers of astroglia (S100β, 21.96%; glial fibrillary acidic protein (GFAP), 18.09%; Fig. 3F, 3G; a fraction of cells coexpressed the two markers, as shown in Supporting Information Fig. 4), of oligodendroglia (2′,3′-cyclic nucleotide 3′-phosphodiesterase, 9.79%; glutathione-S-transferase, 6.25%; NG2, 19.23%, Fig. 3H–3J and Supporting Information Fig. 5; OLIG2, 8.82%; adenomatous polyposis coli (APC), 16.21%; β-tubulin IV, 11.46%; Supporting Information Fig. 5) and of neuronal committment (doublecortin, 19.05% and poly-sialated neural cell adhesion molecule (PSA-NCAM), 18.57%; Fig. 3K and Supporting Information Fig. 5). We did not find GFP+ cells expressing markers of mature neurons (neuronal nuclei, NeuN) or myelinating oligodendrocytes (myelin basic protein, MBP).

### Expression and Activity of the GALC Protein in CNS Tissues of mNSC-Transplanted Twi Mice

**Figure 4:**
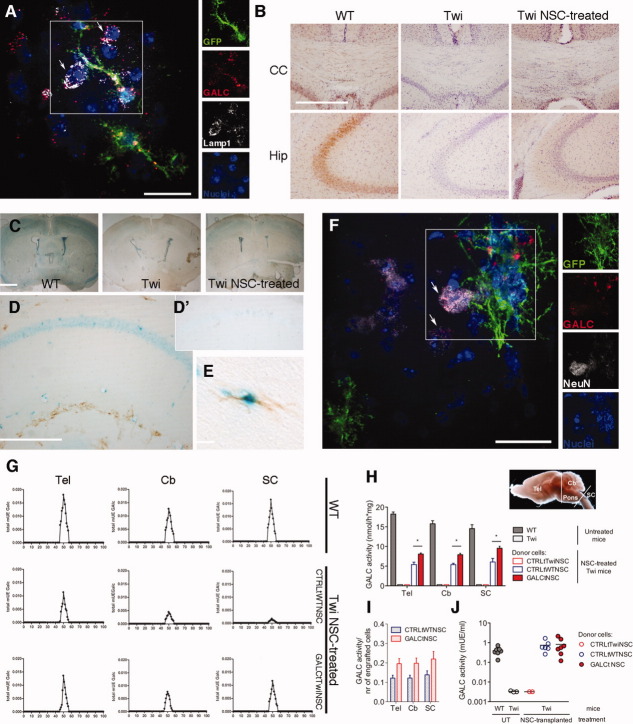
Expression and activity of the β-galactocerebrosidase (GALC) protein in central nervous system tissues of murine neural stem cell (mNSC)-transplanted Twi mice. **(A):** Confocal images (merged picture and single channels of the area highlighted in the inset) following immunofluroscence analysis using anti-GALC, anti-LAMP-1 (lysosomal marker), and anti-GFP antibody in NSC-transplanted Twi brains. The GALC protein is expressed in the lysosomal compartment of GALCtTwiNSC (GFP+ engrafted cells) and GFP-GALC+ cells (putative cross-corrected cells; arrows). Scale bar = 30 μm. **(B):** Expression of GALC (brown) in the corpus callosum and the hippocampus of mNSC-treated Twi mice as well as untreated wild-type (WT) and Twi mice. Scale bar = 500 μm. **(C):** GALC activity (blue, X-Gal) in WT tissues and in NSC-treated compared with untreated Twi mice. **(D, D′):** GALC activity (blue) in brain regions close to engrafted GFP+ mNSC (brown) as well as in regions devoid of engrafted cells **(D)**. **(D′):** Twi tissues show undetectable blue staining. Choroid plexus and blood vessels in WT and Twi tissues showed nonspecific X-Gal staining [42]. **(E):** GALC activity in engrafted mNSC with immature neuronal morphology. Scale bars = 1 mm **(C)**, 500 μm **(D)**, 30 μm **(E)**. **(F):** GFP+ engrafted cells and endogenous neurons (NeuN; arrows) expressing the GALC protein. Confocal merged picture and single channels of the area highlighted in the inset are shown. Scale bar = 20 μm. **(G)** S-300 Gel chromatography showing a peak of enzyme activity in telencephalon (Tel), cerebellum (Cb), and spinal cord (SC) tissues derived from representative untransduced (UT) WT and Twi mice transplanted with CTRLtWTNSC and GALCtTwiNSC. **(H):** Schematic representation of brain dissection. GALC activity in Tel, Cb, and SC tissues of Twi mice transplanted with mNSC expressing physiological or supraphysiological GALC levels. *n* = 12 mice per treatment group except for CTRLtTwiNSC-transplanted mice, in which *n* = 2. Data analyzed by two-way analysis of variance (ANOVA) follow by Bonferroni post-tests. *, *p* < .05 GALCtNSC versus CTRLtWTNSC. **(I):** Total GALC activity normalized by the average number of engrafted cells. **(J):** GALC activity in the cerebrospinal fluid of WT, mNSC-treated, and untreated Twi mice. Data analyzed by one-way analysis of variance (ANOVA) (Kruskal-Wallis *H* test, *p* = .02) followed by Dunnett's multiple comparison test, *, *p* < .05 versus all the other treatment groups. Abbreviations: Cb, cerebellum; CC, corpus callosum; GFP, green fluorescence protein; Hip, hippocampus; SC, spinal cord; Tel, telencephalon.

We next examined whether transplanted mNSC could provide robust levels of the GALC protein to CNS tissues of transplanted Twi mice. Indirect IF using a previously validated antibody to GALC [45] followed by confocal analysis showed GALC expression in the lysosomal compartment (LAMP-1) of engrafted (GFP+) GALCtNSC and of surrounding endogenous (GFP−) cells, suggesting the occurrence of cross-correction (Fig. 4A). This was further confirmed by detection of GALC expression using immunohistochemistry (Fig. 4B). Also, we found GFP- cells with glial and neuronal morphology expressing GALC activity (detected by a histochemical assay [42]; Fig. 4C) in brain regions close to engrafted GFP+ cells as well as in hippocampus and cortex (Fig. 4D, 4E), in which mNSC engraftment was less relevant. Thus, we provided conclusive evidence that engrafted mNSC produce and secrete the GALC protein, which is recaptured and correctly sorted to lysosomes by endogenous host cells, including neurons (Fig. 4F).

By means of S-300 gel filtration chromatography followed by a specific assay for detection of GALC activity [41] we showed a peak of activity corresponding to the MW of the native enzyme (600–700kD) in brain (Tel) and SC tissues derived from mNSC-transplanted Twi mice (Fig. 4G). This peak is absent in tissues from UT Twi mice [19]. The total GALC activity was significantly increased in Tel, Cb, and SC tissues of NSC-transplanted Twi mice with respect to UT Twi controls, reaching consistently higher levels in mice transplanted with GALC-overexpressing cells (45%–65% of WT UT) with respect to mice transplanted with CTRLtWTNSC (30%–40% of WT UT) (Fig. 4H). This was due to higher enzyme expression and secretion by engrafted GALC-overexpressing cells, as assessed by normalizing the total GALC activity for the average number of engrafted NSC in each treatment group (Fig. 4I). No detectable GALC activity was measured in tissues of UT Twi controls or in Twi mice transplanted with CTRLtTwiNSC (Fig. 4H). Brain tissues from GALCtNSC-treated Twi mice analyzed at PND7, PND40, and at terminal stage revealed similar levels of GALC activity (Supporting Information Fig. 3D). This data indicate that early-engrafted mNSC promptly and stably produce and secrete the GALC enzyme.

We previously demonstrated widespread distribution of the GALC enzyme through the CSF flow after direct injection of bdLV.GALC into the Twi CNS [19]. The presence of GALC activity in the CSF of mNSC-treated Twi mice at PND40 (Fig. 4J) confirms this mechanism. The CSF-mediated distribution of the enzyme might also explain the longer time required to achieve stable levels of GALC activity in SC tissues as compared with Tel tissues (Supporting Information Fig. 3D).

### Reduction of Storage and Amelioration of CNS Pathology in mNSC-Treated Twi Mice

The widespread GALC biodistribution observed in mNSC-transplanted Twi mice encouraged us to study the biological effect of the metabolic rescue, trying to assess the impact of GALC-overexpression as compared with expression of physiological GALC levels by donor mNSC. An indicator of the benefit of NSC-mediated enzyme reconstitution is its effect on the major pathological hallmarks of the Twi brain: (a) accumulation of multinucleated monocyte/macrophage-derived globoid cells; (b) microglia activation and astrogliosis, with concurrent upregulation of proinflammatory molecules; (c) oligodendroglial cell death.

**Figure 5:**
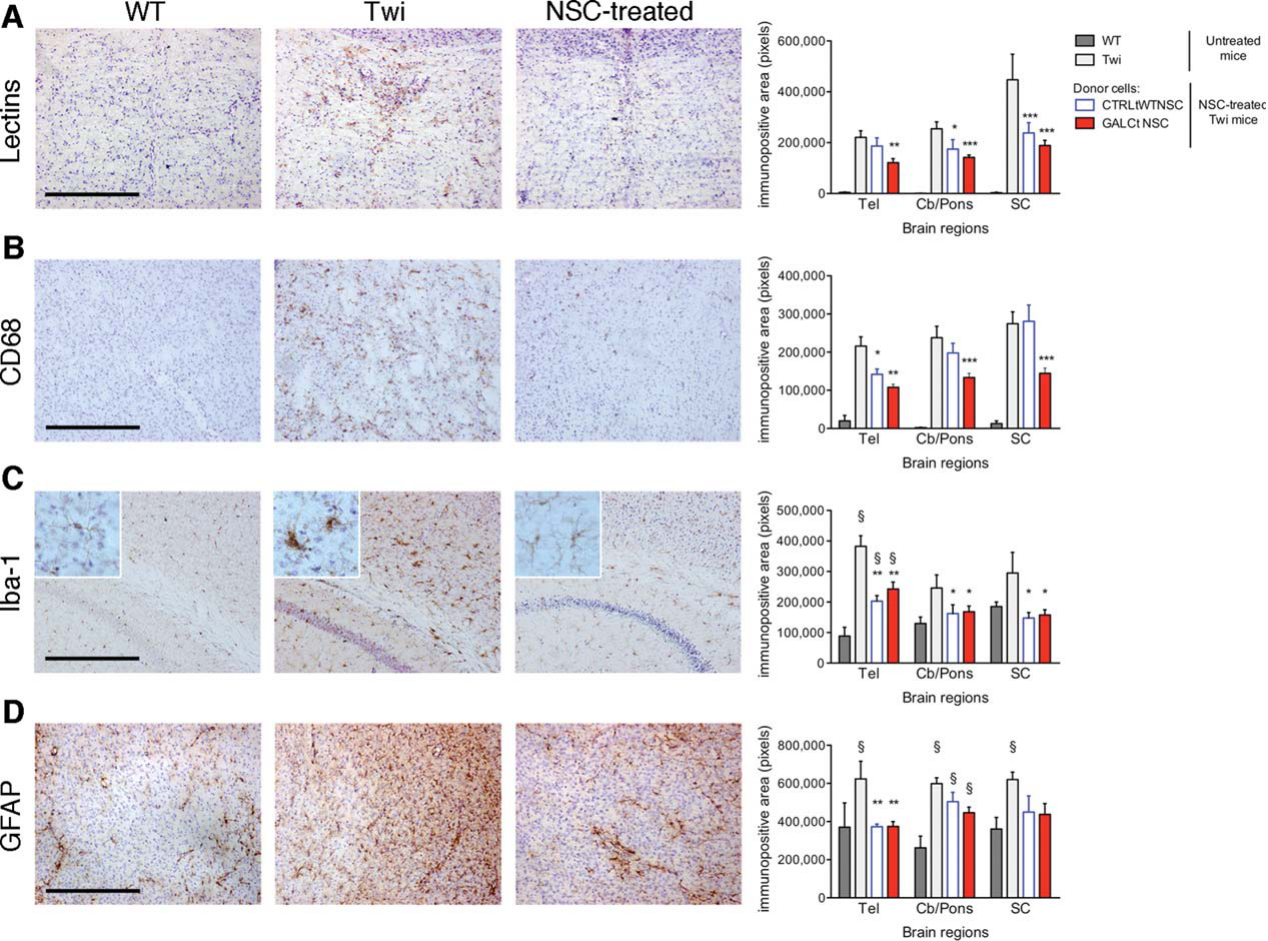
Murine neural stem cell (mNSC) transplants reduce galactolipid storage and ameliorate pathology in central nervous system (CNS) tissues of Twi mice. **(A–D):** Qualitative (representative pictures from different CNS regions: telencephalon [Tel], cerebellum [Cb]/Pons, and spinal cord [SC]) and quantitative analysis of Lectins (**A**; Cb), CD68 (**B**; Tel), Iba-1 (**C**; Tel), and GFAP (**D**; Cb) in tissues from mNSC-treated and untreated wild-type (WT) and Twi mice. Insets in **(C)** show the normalization toward the WT phenotype of Iba-1+ microglial cells in mNSC-treated Twi mice. Scale bars = 1 mm **(A–D)**. *n* = 3–5 mice per treatment group, three slices per brain region. Data analyzed by two-way analysis of variance (ANOVA) followed by Bonferroni post-tests. All groups are significantly different from untreated WT in **(A)** and **(B)** (*p* < .001). §, *p* < .05 versus WT in **(C)** and **(D)**; *, *p* < .05, **, *p* < .01, ***, *p* < .001 versus untreated Twi in **(A–D)**. Abbreviations: GFAP, glial fibrillary acidic protein.

Lectin histochemistry detects galactolipid storage in globoid cells of GLD animal models [46]. We quantified lectin-immunopositive area in selected brain regions (Supporting Information Fig. 6) of mNSC-transplanted and nontransplanted Twi and WT mice. Lectin storage is strongly increased in CNS tissues of untreated Twi mice compared with age-matched WT mice (Fig. 5A). A 30%–50% reduction of tissue storage was detected in the Tel, Cb/Pons and SC of mNSC-transplanted Twi mice as compared with UT Twi controls, with a significative improvement in the Tel of mice transplanted with GALC-overexpressing cells (Fig. 5A).

CD68, Iba-1, and GFAP staining are used to monitor the presence and the activated state of macrophagic, microglial, and astroglial cells, respectively. NSC-transplanted Twi mice showed a significant reduction (30%–40%) of CD68+ cells (Fig. 5B) in regions close to engrafted cells (Tel), thus suggesting NSC-mediated cross-correction and/or immunomodulation. In caudal regions (Cb/Pons and SC), a significant downregulation of CD68 expression was found only in mice transplanted with GALC-overexpressing mNSC. A significant reduction of Iba-1 expression (Fig. 5C) and normalization of the cell morphology (insets in Fig. 5C) were found in all the CNS regions of mNSC-treated Twi mice. Downregulation of GFAP expression (Fig. 5D) was present in the Tel and, to a lesser extent, in the Cb/Pons and SC of mNSC-treated Twi mice.

Twi mice display a mild and patchy demyelination in the telencephalic white matter (Kluver-Barrera staining; Supporting Information Fig. 7A). In addition, fewer glutathione S-transferase (GST)-π+ cells with the typical oval cytoplasm and a general disarrangement of these cells are present along the CC of Twi mice, together with scattered GST-π+ debries, possibly fragmented myelin and oligodendrocyte processes (Supporting Information Fig. 7B–7D). The myelin appearance, the morphology, and the organization of GST-π+ cells in the CC were ameliorated in mNSC-treated Twi mice with respect to untreated Twi controls (Supporting Information Fig. 7C, 7D).

**Figure 6:**
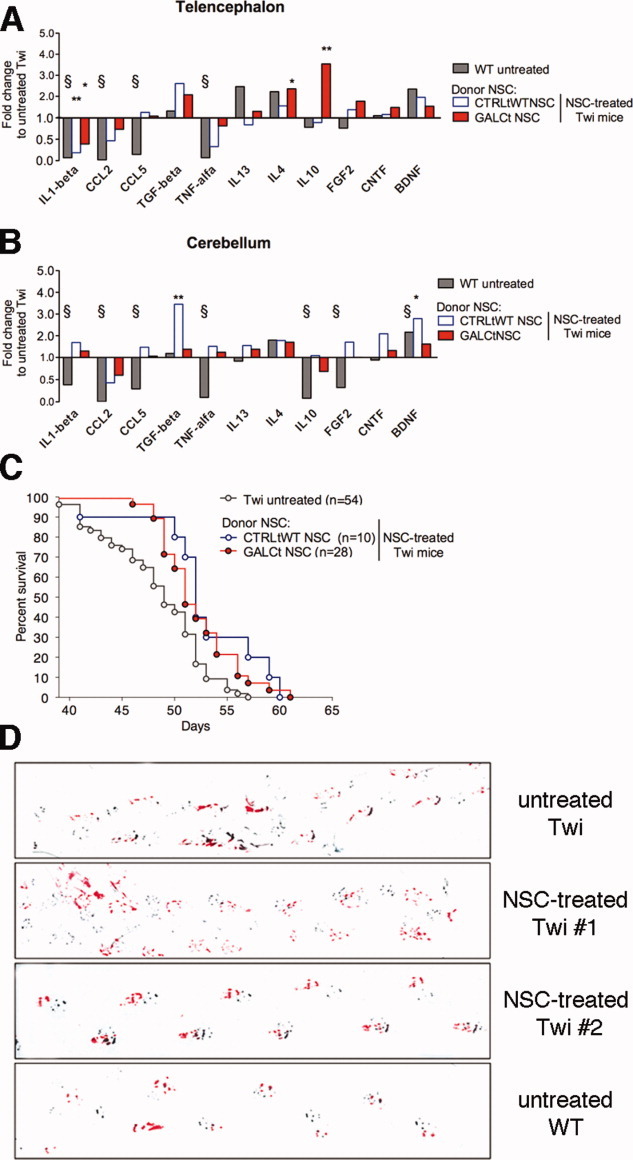
Transplanted murine neural stem cells (mNSC) decrease inflammation, delay the onset of symptoms, and improve motor function of Twi mice. **(A, B):** mRNA expression of inflammatory markers in telencephalon **(A)** and cerebellum **(B)** tissues from wild-type (WT) and mNSC-treated mice expressed as fold change over the Twi levels. All samples were normalized to β-actin content. *n* = 3–4 mice per treatment group. Data analyzed by two-way analysis of variance (ANOVA) followed by Bonferroni post-tests. *, *p* < .05, **, *p* < .01 versus untransduced (UT) Twi. §, *p* < .05 UT WT versus UT Twi. **(C):** Kaplan-Meier survival curves of NSC-treated and untreated Twi mice. Log-rank (Mantel-Cox) test, *p* = .0061 (GALCtNSC), and *p* = .0037 (CTRLtWTNSC) versus untreated Twi. GALCtNSC versus CTRLtWTNSC, *p* > .05. **(D):** Gait analysis performed at postnatal day 40 (footprints: black, front paws; red, rear paws). Number of mice analyzed: NSC-treated, *n* = 25 (*n* = 18 GALCtNSC and *n* = 7 CTRLtWTNSC); untreated WT, *n* = 9; untreated Twi, *n* = 7. Twi #1 and Twi #2 are representative examples of GALCtNSC-treated Twi mice.

As mNSC have an anti-inflammatory activity when transplanted into a chronic inflammatory [47], ischemic [48], or traumatized CNS environment [49], we sought to assess whether a similar activity could be displayed also in Twi CNS, which is characterized by extensive neuroinflammation at late stages of disease progression [50]. We performed Syber-green quantitative polymerase chain reaction on mRNA isolated from the Tel and Cb of mNSC-treated Twi mice at PND40, as well as from age-matched UT WT and Twi littermates. We checked for several proinflammatory chemokines and cytokines known to be upregulated in the Twi CNS compared with WT CNS [i.e., interleukin (IL)-1β, MIP1-α, tumor necrosis factor-alfa (TNF-α), and chemokine (C-C motif) ligand 2 (CCL2)] as well as for molecules that could exert an anti-inflammatory (IL-4 and IL-10) or neuroprotective effect [brain derived neurotrophic factor (BDNF) and ciliary neurotrophic factor (CNTF)]. Our results showed that mRNA levels of the proinflammatory cytokine IL-1β, of the monocyte chemoattractant CCL2 and of TNF-α were downregulated in mNSC-transplanted as compared with untreated Twi mice, both in the Tel (Fig. 6A) and in the Cb (Fig. 6B). Interestingly, IL-10, IL-4, and BDNF mRNA levels were upregulated by mNSC treatment, suggesting an immunomodulatory and neuroprotective role for mNSC in the Twi environment (Fig. 6A, 6B) that is better appreciable in the Tel, the region with the highest density of engrafted cells.

Overall, these data demonstrated that mNSC transplantation in Twi mice results in clearance of tissue storage and amelioration of histopathology, also suggesting a positive correlation between therapeutic efficacy and supranormal GALC expression in donor cells. In addition to or as a consequence of enzyme secretion, we observe an overall reduction of CNS inflammation and the upregulation of growth factors that might contribute to counteract tissue damage.

### Injection of mNSC Delays the Onset of symptoms, Prolongs Survival, and Ameliorates Motor Function of Twi Mice

We next examined whether the partial but significant impact of mNSC transplantation in reducing the major pathologic hallmarks correlated with functional rescue, assessed as prolonged survival, and improved motor function. mNSC-treated Twi mice showed a delay of 6–7 days in the onset of the symptoms. The average lifespan (days) of Twi mice transplanted with CTRLtWTNSC and with GALCtNSC (either Twi or WT) was 52.7 ± 1.7 (*n* = 10) and 51.3 ± 0.6 (*n* = 28), respectively (*p* > .05). Both values were significantly different from the average lifespan of UT Twi mice (47.6 ± 0.7; *n* = 54). The survival curves of mNSC-treated differed significantly from that of UT Twi mice (Fig. 6C). Importantly, a significant increase in cumulative survival was observed between PND40 and PND50 in the mNSC-treated group (i.e., cumulative survival at PND49 was 80.0 ± 12.6, 71.4 ± 8.5, and 46.3 ± 6.7 for CTRLtWTNSC-treated, GALCtNSC-treated, and UT Twi mice, respectively; *p* < .05 NSC-treated vs. UT Twi; *p* > .05 CTRLtWTNSC-treated vs. GALCtNSC-treated).

At PND40, Twi mice display a severe twitching and are highly compromised in their walking ability, due to almost complete paralysis of hind limbs (Fig. 6D; UT Twi). The walking performance was improved in the majority of NSC-treated mice as compared with UT Twi littermates, with some mice behaving similar to WT controls (17/25 mice analyzed, 68%; four separate transplantation experiments; footprints of two representative mice, #1 and #2, are shown; Fig. 6D). No obvious differences were found between mice transplanted with CTRLtWTNSC or GALCtNSC. All together these results indicate that rescue of GALC activity achieved by mNSC therapy in the brain of Twi mice provides amelioration of CNS histopathology and results in delayed onset of symptoms, improved survival and motor function. However, death eventually occurs, likely consequent not only to the inability of NSC treatment to target the PNS but also to the inadequacy of the GALC activity supply provided by engrafted NSC (which is stable over time) to counteract the build up of intracellular storage at the late stages of disease progression, particularly in the caudal CNS regions that rely exclusively on GALC transport and cross-correction to achieve metabolic correction. Indeed, a partial clearance of lectin storage is still detected in the Tel but not in the Cb/Pons of Twi mice transplanted with GALC-overexpressing NSC and analyzed at the terminal stage (Supporting Information Fig. 8).

### Engraftment and Enzymatic Correction of mNSC Are Shared by hNSC

**Figure 7:**
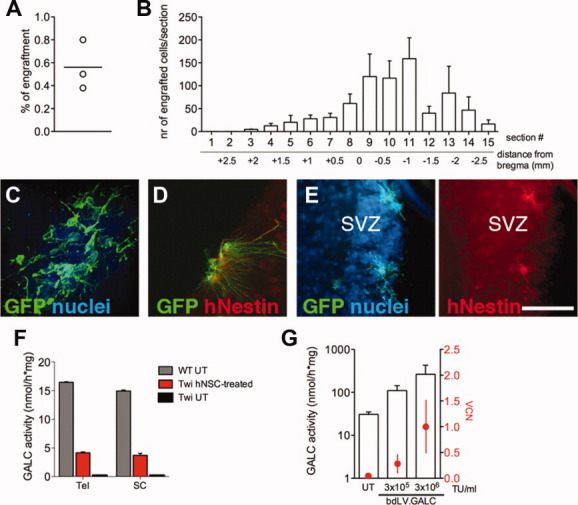
Cell engraftment, distribution, and metabolic correction of human neural stem cell (hNSC) in Twi mice. **(A):** Percentage of hNSC engraftment. **(B):** Distribution of engrafted hNSC (*n* = 3 mice). **(C–E):** hNSC engrafted in the forebrain periventricular region of Twi brains after immunofluroscence analysis using antibodies to GFP and to human nestin. Scale bars = 250 μm (shown in **E**). **(F):** β-galactocerebrosidase (GALC) activity in central nervous system tissues (telencephalon and spinal cord) of hNSC-transplanted Twi mice (*n* = 3). **(G):** GALC overexpression in hNSC following bdLV.GALC mediated gene transfer. *n* = 3 independent experiments. Abbreviations: GFP, green fluorescence protein; SC, spinal cord; SVZ, subventricular zone; Tel, telencephalon; TU, transforming units; UT, untransduced; VCN, vector copy number; WT, wild-type.

Somatic NSC derived from the human fetal brain and cultured as neurospheres [39, 51] physiologically produce the GALC protein, showing levels of enzymatic activity comparable with that measured in WT mNSC (33.67 ± 4.48 nmol/hour/mg; compare with Fig. 1A). We transplanted hNSC transduced with bdLV.CTRL in the brain ventricles of newborn Twi mice. Cell engraftment (Fig. 7A) and distribution (Fig. 7B) assessed at PND40 were remarkably similar to those described in mNSC-transplanted Twi mice, suggesting low immunogenicity of hNSC xenograft. Engrafted hNSC displayed immature morphology and immunophenotype (Fig. 7C–7E), the vast majority of them expressing nestin (Fig. 7D, 7E). They produced and secreted the GALC protein, allowing partial restoration of enzyme activity (20%–25% of WT levels), in Tel and SC tissues of transplanted mice (Fig. 7F). These results, together with data indicating that efficient LV-mediated overexpression of GALC (Fig. 7G) is achievable in hNSC, support the feasibility and therapeutic potential of hNSC gene therapy for the treatment of GLD.

## DISCUSSION

In this study, we showed that intracerebroventricular NSC transplantation provides rapid, robust, and long-lasting NSC engraftment and production of the GALC enzyme in CNS tissues of Twi mice, a severe model of GLD. We obtained proof of principle for this approach with murine and hNSC. We clarifyed that several mechanisms (enzyme secretion and distribution, modulation of inflammation, neuroprotection, and cell replacement) concur to achieve NSC-mediated benefit and elucidated the impact that supranormal enzyme levels in donor cell might have on the pathology. Overall, we comprehensively addressed some significant and still controversial issues regarding gene/NSC-based approach to treat GLD, highlighting the potential advantages of this strategy as well as its drawbacks.

The extent of donor/host cell chimerism required to achieve therapeutic benefit in diseases characterized by disseminated CNS damage, the ideal balance between differentiation and persistence of stem/progenitor cells into the targeted tissue and the ideal mechanism of tissue repair to foster (whether it has to be cell replacement or tissue protection) are key and still unsolved issues. The delivery of NSC in the cerebral ventricles of newborn mice described here allowed a relatively uniform rostrocaudal distribution of the cells, which rapidly accessed the SVZ, the forebrain white matter tracts (along which they also migrated) and the hippocampal region, while the most caudal regions were less effectively engrafted. In line with previous reports [24, 32], we showed that less than 3% of the injected NSC (regardless the cell preparation used) engraft and survive in the host CNS (either Twi or WT, thus excluding a preferential tropism due to the disease). Interestingly, doubling the number of injected NSC did not result in increased engraftment, suggesting the presence of a tight regulation as regard to the number of exogenous cells that the host brain can integrate, even if the injection is performed in neonates as compared with adults [24].

The inflammatory environment that characterizes Twitcher CNS tissues at the late stages of disease progression has been proposed as a major cause for the limited survival of NSC transplanted in early postnatal Twitcher brains, which resulted in progressive decrease of enzyme expression [31]. The stable number of engrafted cells that we found throughout the brain of transplanted Twi mice at PND7, PND40, and even at terminal stage indicates the environment in the Twi brain is permissive for the engraftment and long-term survival of NSC, thus implying that NSC-based strategies could be feasible in GLD. In addition, we showed that WT or enzyme-overexpressing NSC do not have a significant advantage in terms of engraftment and survival over Twi cells, suggesting that intrinsic NSC resistance to psychosine [33] might not be strictly related to the levels of GALC expression.

Approximately 50% of long-term engrafted NSC expressed markers for mature astrocytes, oligodendroglial cells and neuronal progenitors. The absence of NSC-derived mature neurons and myelinating oligodendrocytes might implicate that additional cues or additional time are required to achieve full maturation of these cell types. However, this apparent lack of terminal differentiation and the propensity of maintaining an undifferentiated phenotype within the host tissue support recent preclinical data indicating that transplanted NSC are therapeutic efficacious in models of neurodegenerative diseases via a number of bystander mechanisms alternative to the expected cell replacement [14, 24, 27, 48, 51, 52].

In line with this view, we showed that NSC behaved not only as effective pumps for the deficient enzyme but also displayed putative immunomodulatory and neuroprotective functions upon transplantation in the Twi brain. The total GALC activity in the CNS of transplanted Twi mice reached 50% the WT level, not only in the telencephalon, in which we found the majority of engrafted cells, but also in the cerebellum and in the SC. Widespread enzyme distribution and cross-correction underlie the therapeutic activity ensured by NSC transplantation, as they are for the direct intracerebral gene delivery approach [12, 13]. Using several complementary assays, we conclusively demonstrated that the GALC enzyme secreted by NSC is transported through the CSF and is recaptured by endogenous cells. Cross-correction is particularly efficient in neurons following direct intracerebral delivery of LV.GALC and LV.ARSA in mutant mice [19, 38], likely reflecting a prevalent neuronal localization of the M6PR [53, 54]. Still, the presence of cross-corrected oligodendrocytes and astrocytes in the brain of LV.GALC-treated Twi mice [19] and the results of in vitro experiments reported here suggest the occurrence of M6PR-independent uptake of the GALC enzyme in neural cells. Several molecules and mechanisms have been implicated in M6PR-independent transport of most acid hydrolases [44]. Further investigation on the mechanisms of GALC uptake in neural cells would be of importance for improving gene/cell therapies aimed to treat GLD.

NSC-transplanted Twi mice showed decreased brain levels of mRNA species (IL-1β, TNF-α, MIP-1α) known to be produced by activated macrophages/microglia and astroglia and able to promote and sustain inflammation in the Twi brain [50]. Our data do not allow dissecting the contribution of a by-stander effect of NSC with respect to the activity of the GALC enzyme they produced. However, the marked downregulation of IL-1β and TNF-α in the telencephalon as compared with the cerebellum, the upregulation of anti-inflammatory molecules (IL-10) and of neurotrophic factors (BDNF) detected in CNS tissues of NSC-transplanted Twi mice suggest that, besides providing the missing enzyme, NSC might have neuroprotective and immunomodulatory functions in the context of reciprocal donor-host signaling in the Twi environment.

Our data strongly suggest that clearance of glycolipid storage in macrophages and downregulation of activated microglia and astroglia correlate with the levels of GALC activity in CNS tissues of transplanted mice. These, in turn, correlate with levels of GALC activity in donor NSC. While NSC expressing physiological GALC levels might provide the enzymatic activity required to normalize several pathological hallmarks in the high-density engrafted regions, the presence of GALC-overexpressing NSC is required to provide therapeutically relevant GALC levels in the cerebellum and in the SC. Thus, similar to what observed in vitro, LV-mediated GALC overexpression results in more efficient enzyme secretion in the extracellular space and, likely, in higher availability of cross-correcting enzyme in regions distant from the site of preferential NSC engraftment.

The metabolic correction provided by NSC transplants resulted in a functional benefit in Twi mice. Indeed, NSC-treated Twi mice in this study showed a significant increase in survival proportions between PND40 and PND50, the period in which 80% of untreated Twi mice die, clearly indicating that NSC treatment delays the onset of symptoms. The overall improved conditions are further confirmed by the amelioration of walking ability observed at PND40, the time at which we assessed histopathology and inflammatory profile.

The gain in survival of NSC-treated Twi mice in this study was lower than that obtained by conventional HCT [55, 56] or in combined approaches [56, 57] but similar to that obtained by AAV.GALC-mediated intracerebral gene transfer [58, 59], intrathecal enzyme replacement therapy (ERT) [60] and transplantation of neural progenitors [31] or mesenchymal-lineage stem cells [61], the latter sharing with NSC the immunomodulatory and anti-inflammatory activity [62, 63] but displaying a poor yield of long-term engraftment upon neonatal intracerebral transplantation in the Twi brain [61, 64]. Overall, the functional amelioration of NSC-treated Twi mice shown here is moderate if compared with the improvement in pathology and ultimately animals succumb to the disease.

Also, while the potential therapeutic advantage of above-normal enzyme expression in donor cells has been demonstrated for HSC gene therapy in MLD [8], MPS I [65], and GLD mouse models [66], in this study Twi mice transplanted with GALC-overexpressing NSC did not show a significant amelioration in terms of survival as compared with Twi mice transplanted with NSC expressing physiological enzyme levels. Several issues related to the biology of disease and of the donor cells might be considered to explain these apparent contradictory results. First, clearance of storage strictly correlates with the availability of functional enzyme in the mutant cells, but the threshold of enzyme activity required at a single cell level to achieve therapeutic benefit is not defined. Our comparative analysis performed on NSC-treated Twi mice at PND40 and at terminal stage strongly suggests that this threshold increases during the disease progression. Thus, even the supply of GALC activity provided by engrafted enzyme-overexpressing NSC is likely inadequate to counteract the rapid increase of intracellular storage, particularly in the caudal CNS regions, which are affected earlier during the disease progression as compared with the forebrain. Second, multiple mechanisms contribute to create symptomatology in Twi mice, ultimately determining their survival. In particular, the severe PNS demyelination, which is not targeted by NSC transplants, strongly contributes to the sharp decrease in cumulative survival observed in Twi mice, irrespectively of treatment, from PND45 onward. Third, it has been proposed that GALC deficiency induces a psychosine-dependent neuron-specific damage before or simultaneously to demyelination [67–69]. This might further explain the limitation of CNS-directed gene and cell terapies, including NSC and HCT-based treatments, to treat GLD.

Several preclinical studies indicate low immunogenicity and virtually no tumorigenicity of somatic hNSC following transplantation in the neonatal and adult rodent CNS [21, 27, 70–72]. The low immunogenic potential of hNSC that we describe here in immunocompetent Twi mice is likely due to the privileged immunological condition of the neonatal brain and the capacity of hNSC to crosstalk with the inflamed CNS [14, 51]. These data provide additional rationale to the development of allogenic hNSC-based approach, which is currently the only feasible option in humans [73]. Safe gene transfer [22, 51, 74] and GALC overexpression (our unpublished results) can be achieved in hNSC. However, the clinical development of NSC gene therapy poses an additional layer of complication when compared with NSC therapy alone. Thus, careful preclinical studies in the relevant animal models are required to assess the long-term safety and the therapeutic advantage of above-normal enzyme expression in donor hNSC.

## CONCLUSION

Future therapeutic intervention for complex diseases like GLD and similar LSD, particularly for the severe infantile forms, will likely require multidisciplinary strategy and multifaceted approaches. Combination of CNS-directed therapies, such as intracerebral gene delivery [19, 59] or NSC gene therapy [33; this study] with therapies targeting the PNS, such as conventional HCT [7, 10] or novel and more efficient HSC gene therapy strategies [75] performed in the early PNDs of life may hope to achieve rapid and sustained levels of the functional enzyme to the affected tissues in an appropriate time-window, thus consistently prolonging life of affected patients.
