# Supplementary figures and images for: Neural Stem Cell Gene Therapy Ameliorates Pathology and Function in a Mouse Model of Globoid Cell Leukodystrophy
Source: Stem Cells. 2011 Aug 1;29(10):1559–71. doi: 10.1002/stem.701 (PMC3229988; doi:10.1002/stem.701)

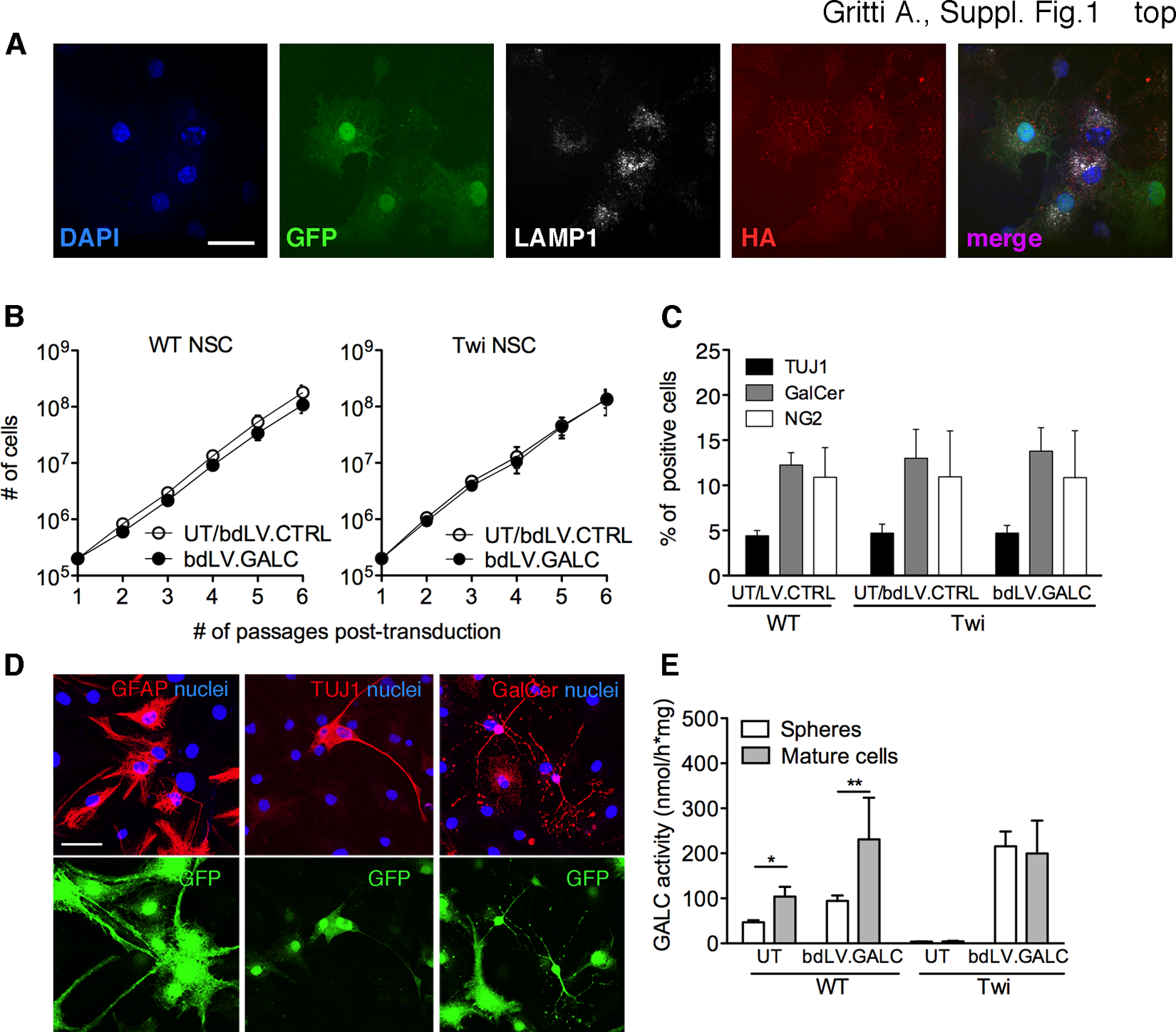

Supplement: Supplementary file 1 [file stem0029-1559-SD1.tif]

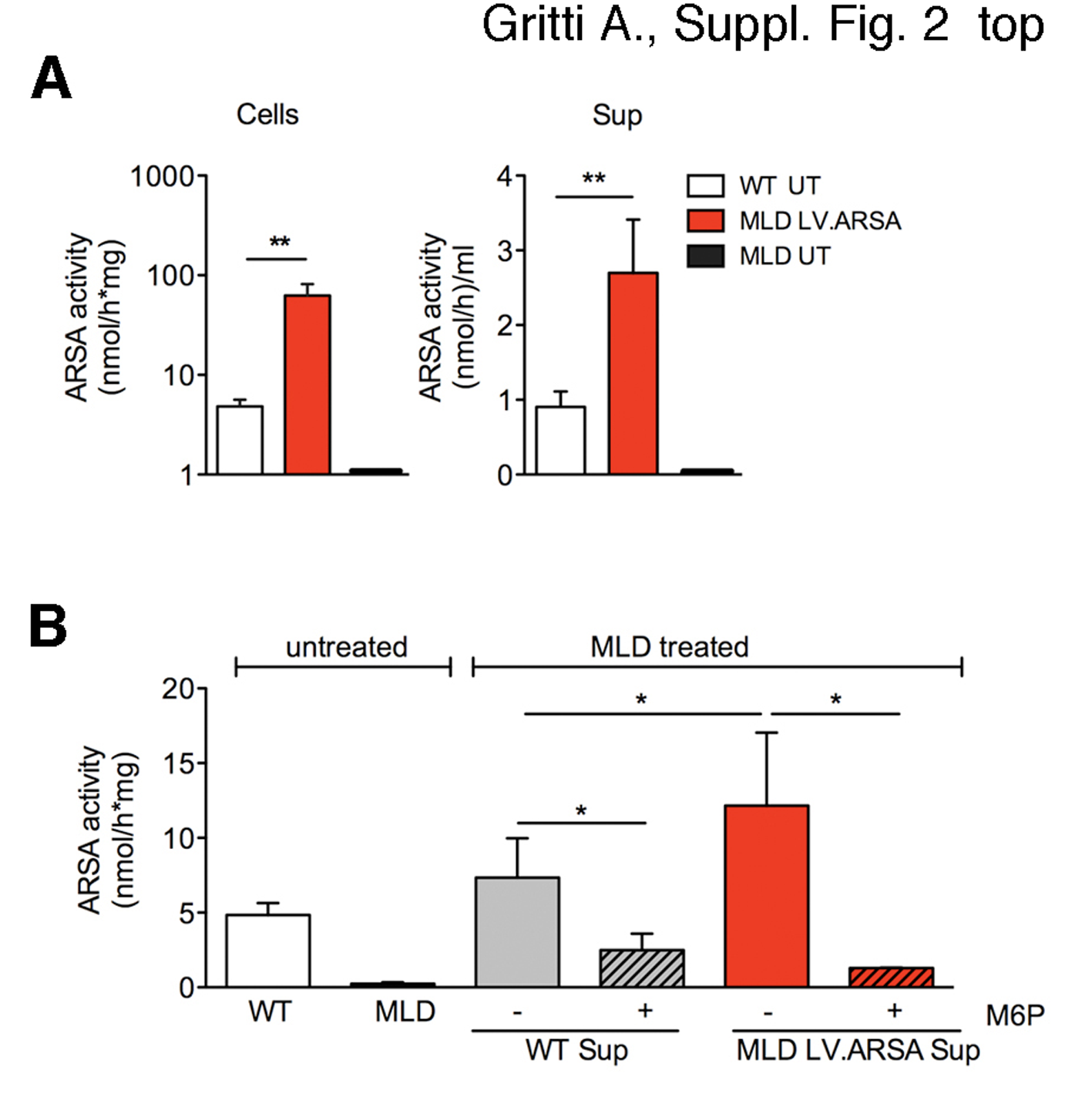

Supplement: Supplementary file 2 [file stem0029-1559-SD2.tif]

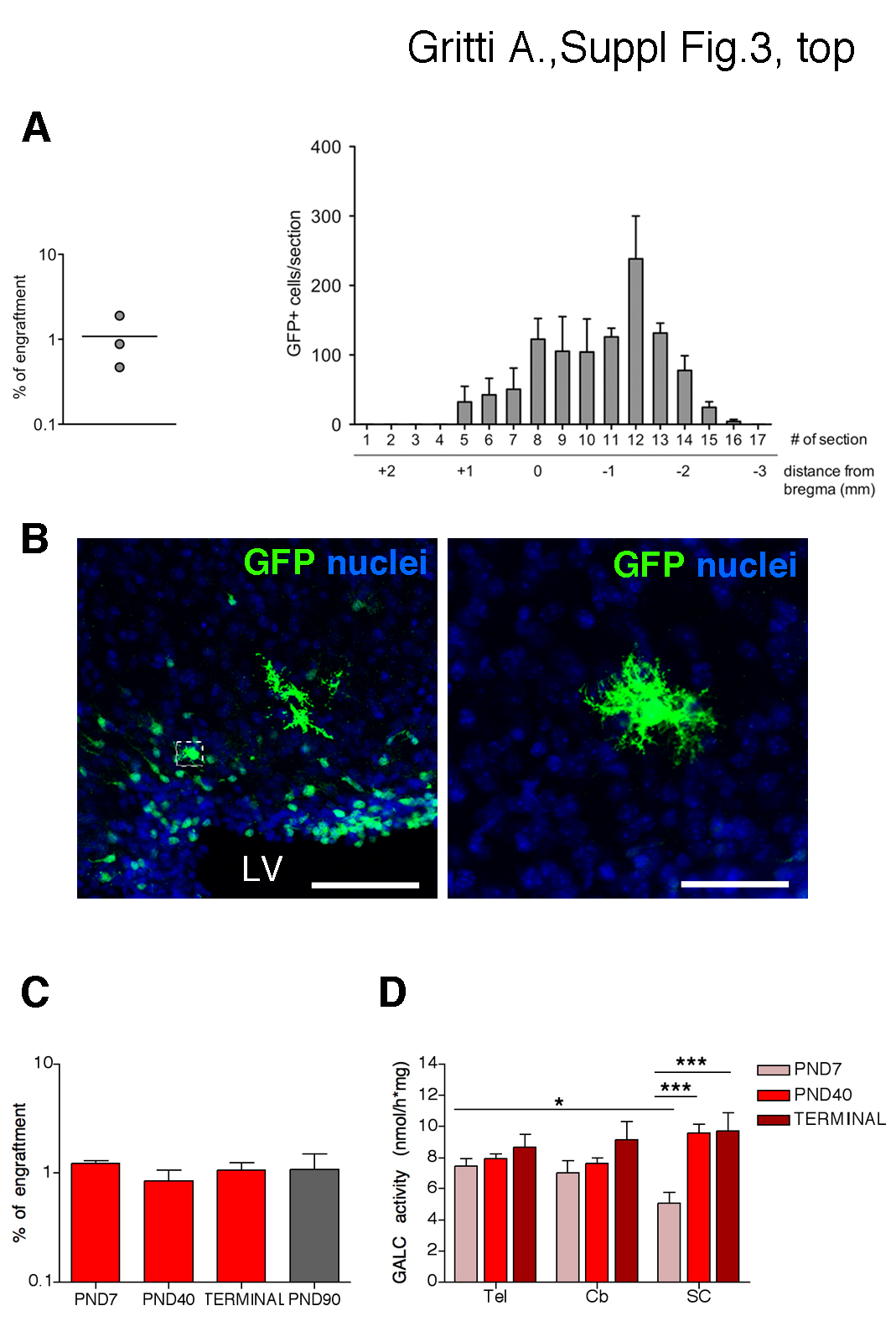

Supplement: Supplementary file 3 [file stem0029-1559-SD3.tif]

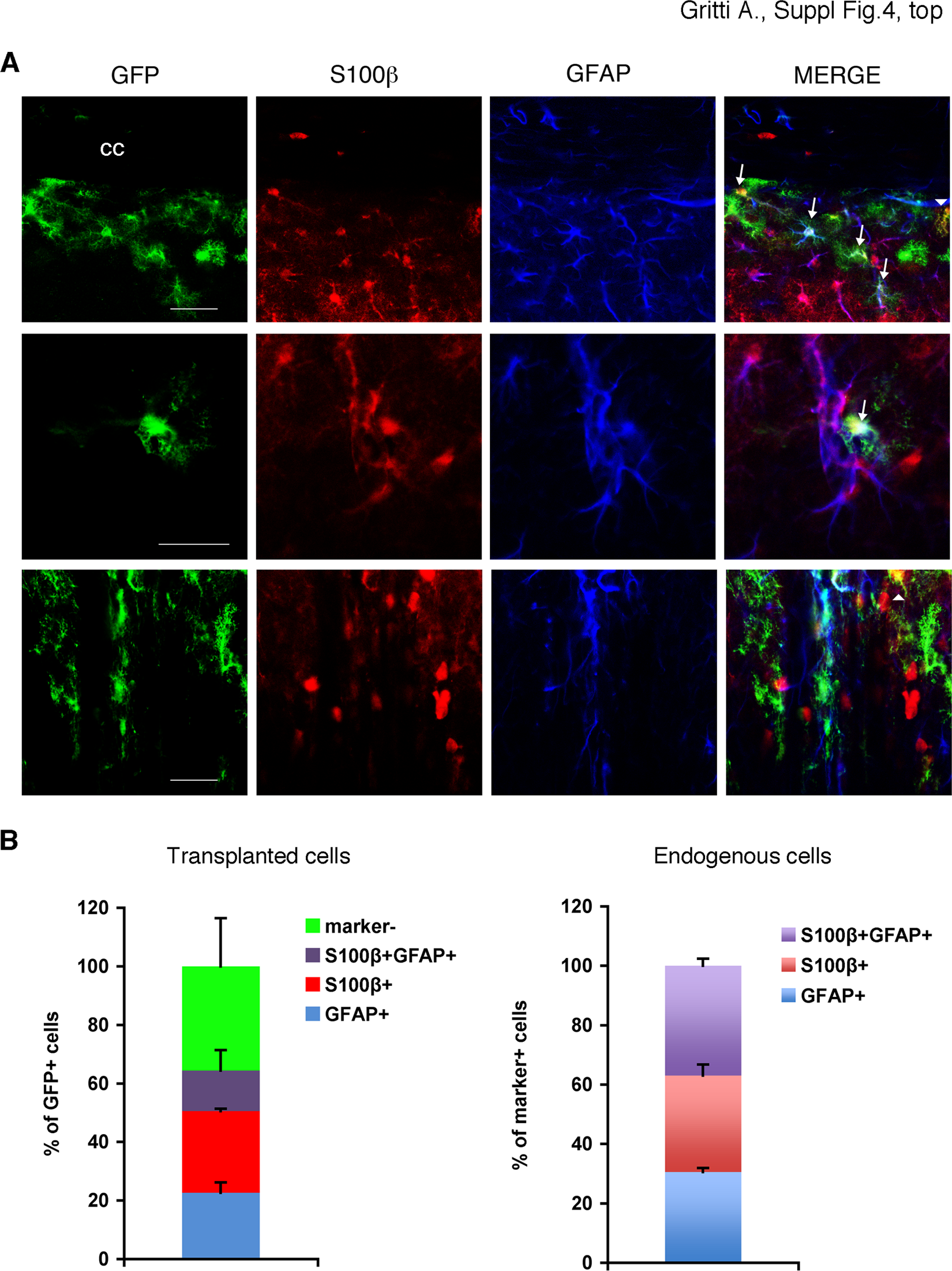

Supplement: Supplementary file 4 [file stem0029-1559-SD4.tif]

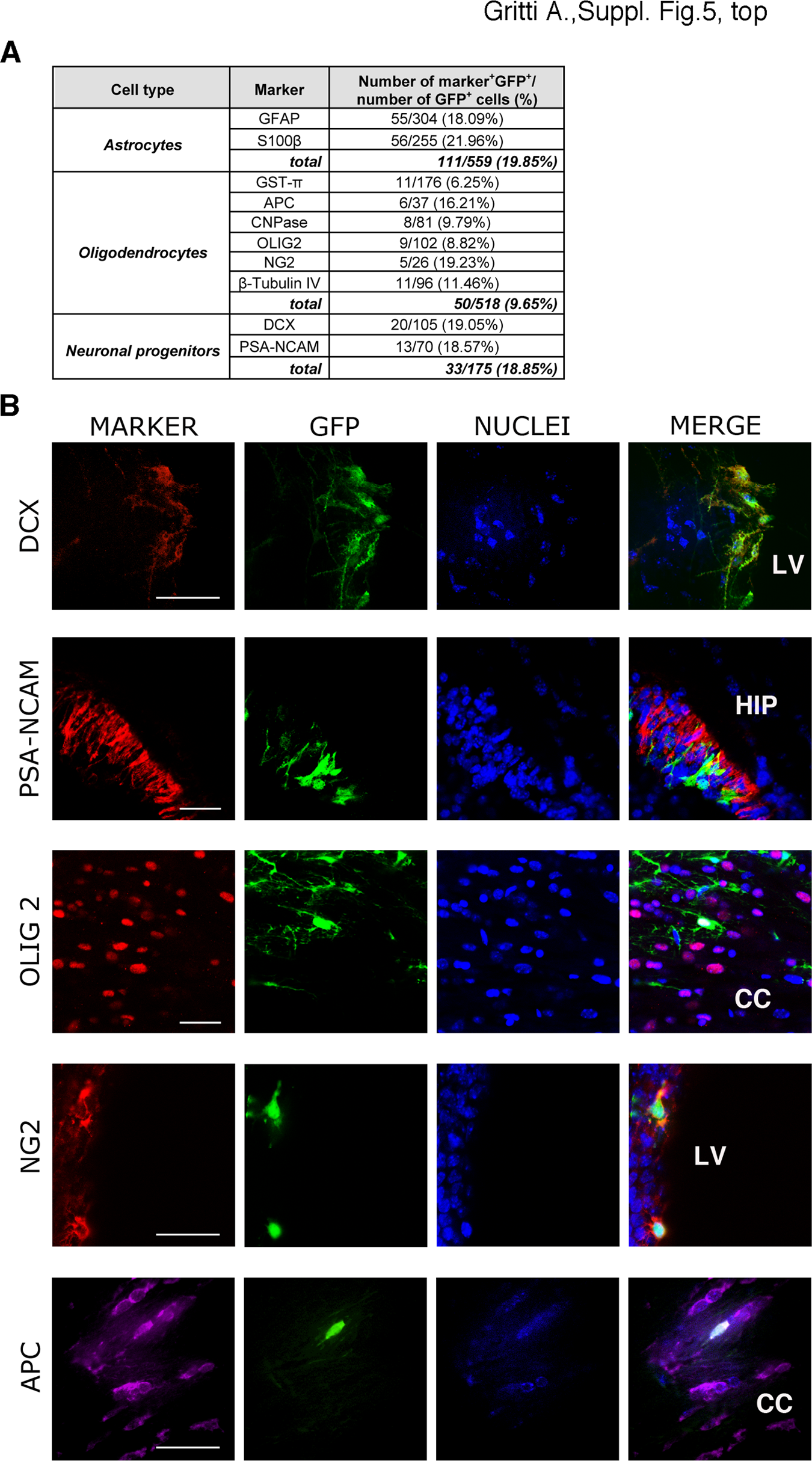

Supplement: Supplementary file 5 [file stem0029-1559-SD5.tif]

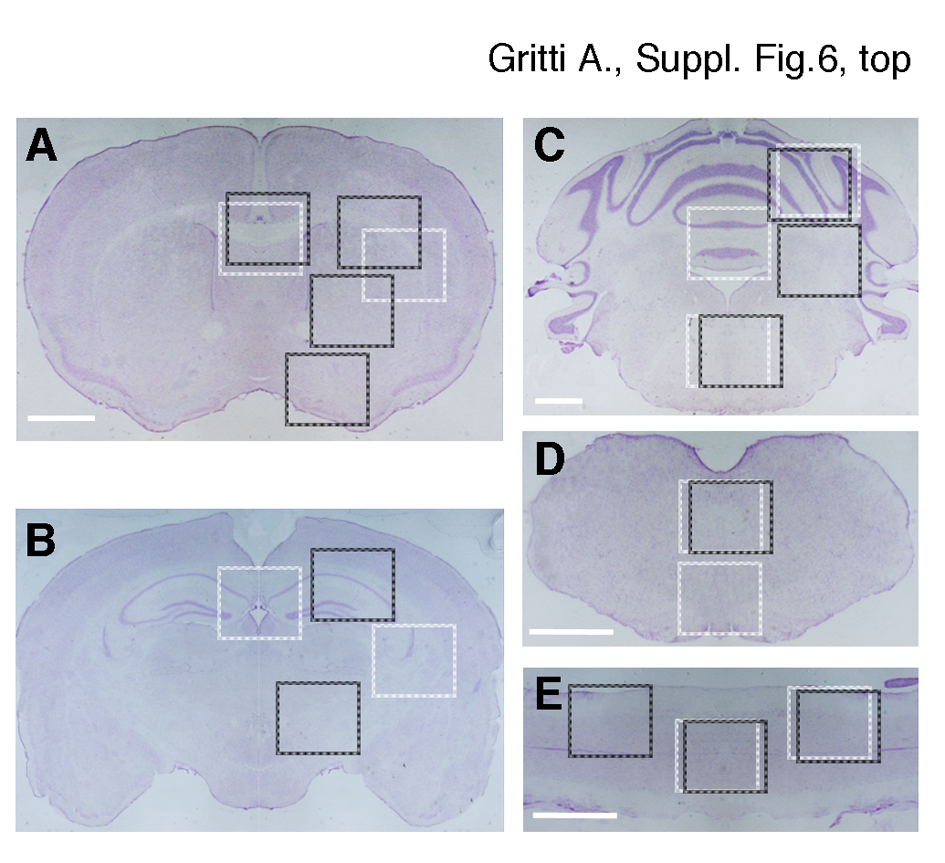

Supplement: Supplementary file 6 [file stem0029-1559-SD6.tif]

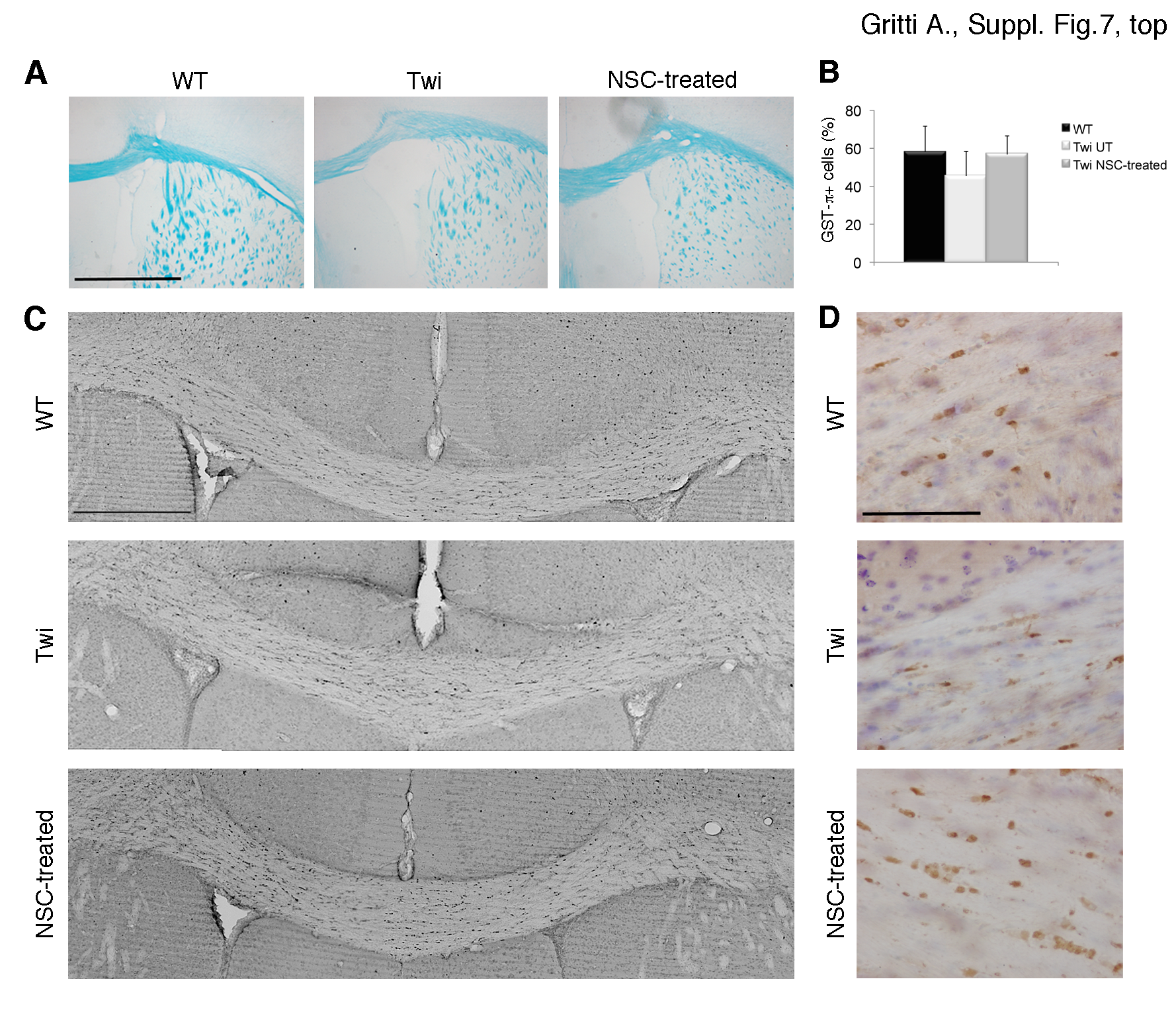

Supplement: Supplementary file 7 [file stem0029-1559-SD7.tif]

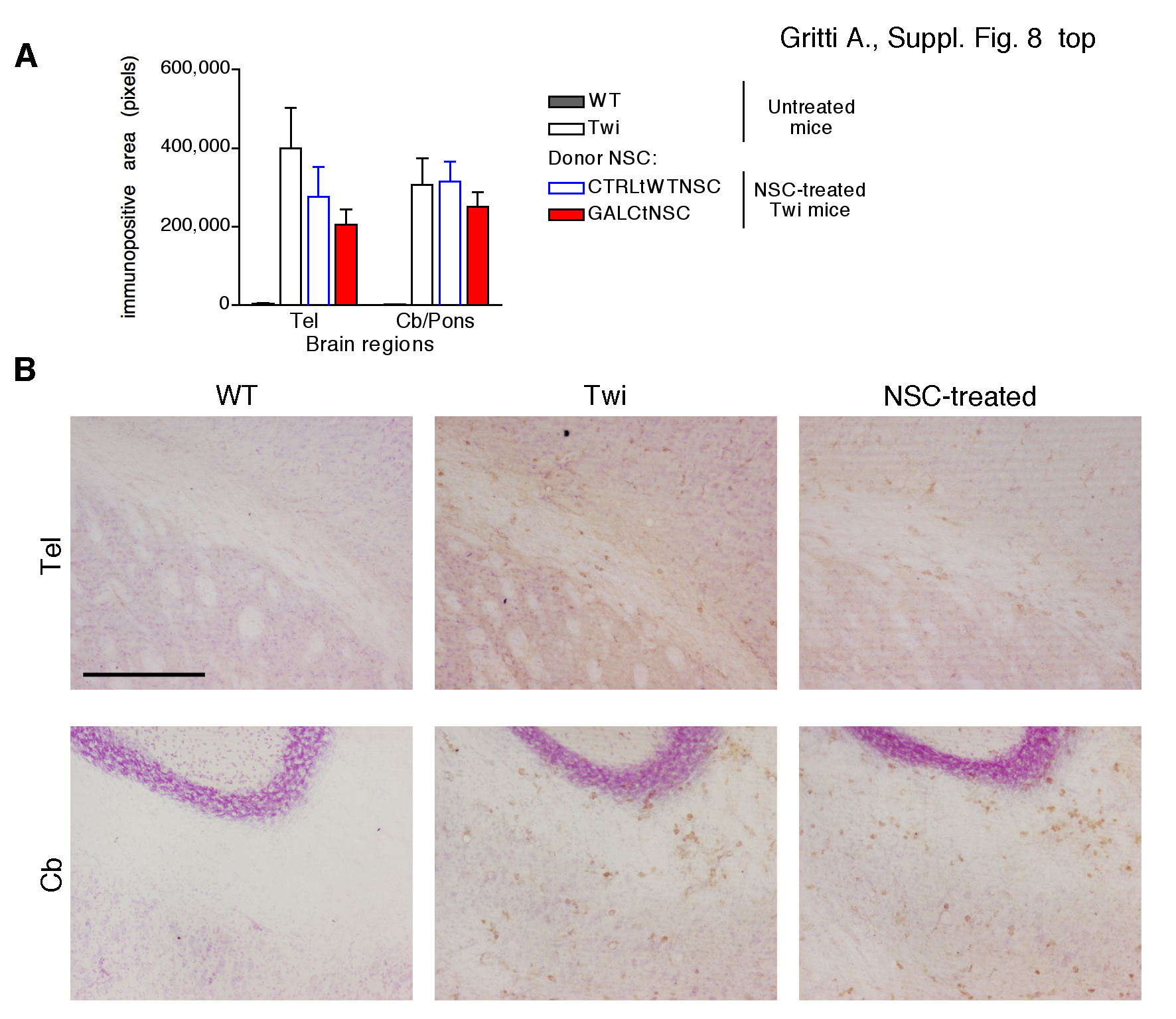

Supplement: Supplementary file 8 [file stem0029-1559-SD8.tif]

**Supplementary Table 2. Experimental groups of animals used for the different assays.**


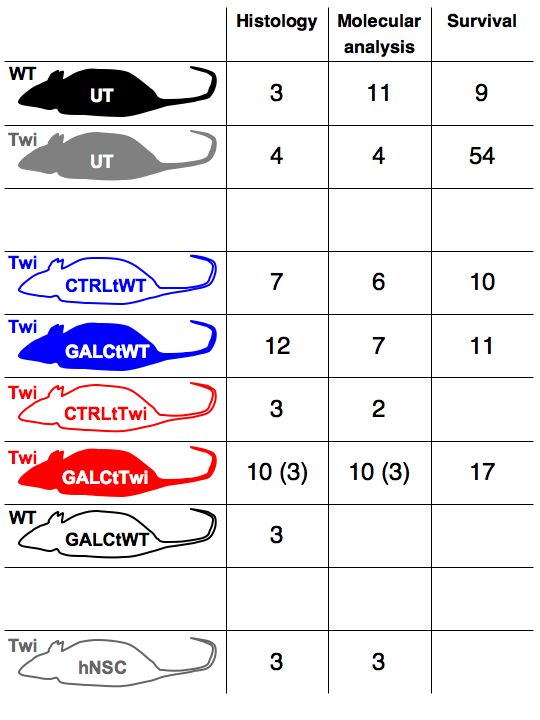

Supplement: Supplementary file 11 [file stem0029-1559-SD11.doc]
