# Supplementary material for: Neural Stem Cell Gene Therapy Ameliorates Pathology and Function in a Mouse Model of Globoid Cell Leukodystrophy
Source: Stem Cells. 2011 Aug 1;29(10):1559–71. doi: 10.1002/stem.701 (PMC3229988; doi:10.1002/stem.701)
Supplement: Supplementary file 9 [file stem0029-1559-SD9.doc]

**Supplemental Experimental Procedures**

**Lentiviral vector preparation and titration**

VSV-pseudotyped third-generation LV were produced by transient four-plasmid cotransfection into 293T cells and purified by ultracentrifugation as described [1], with the modification that 1M sodium butyrate was added to the cultures for vector collection. Expression titer of vectors was estimated on HeLa cells by limiting dilution. Vector particles were measured by HIV-1 gag p24 antigen immunocapture (NEN Life Science Products). Concentrate vector expression titres were 2-3x10^9^ TU/ml and infectivity was 2-6x10^4^ TU/ng of p24 for all LV preparations.

**NSC isolation, propagation and transduction**

**mNSC.** Neonatal (PND2) Twi or MLD mice and age-matched WT littermates were anaesthetised by placing them in crushed ice for 4 min. Brains were removed and tissue containing the subventricular zone (SVZ) of the forebrain lateral ventricles was dissected out. Tissues from 3 mice were pooled to establish a culture (*n=*2-3 independent cultures).

**hNSC.** We used a NSC line established and propagated from the diencephalic/telencephalic brain region of a human (h) foetus at 10.5-week gestational age, according to previously described protocols [2]. Brain foetal tissue obtained from Advanced Bioscience Resources, Inc., Alameda, CA, USA. The San Raffaele Scientific Institute Ethical committee approved the use of human foetal tissue for the establishment of continuous non-transformed hNSC lines (protocol HPCT).

LV transduction was performed on serially subcultured NSCs (passages 5-10 and passages 18-25 for mNSC and hNSC, respectively). Cells were incubated overnight with concentrations of vector preparations: 3x10^7^ TU/ml (mNSC); 3x10^5^ and 3x10^6^ TU/ml (hNSC). LV-containing medium was then removed and neurospheres were subcultured every 5-10 days by mechanical dissociation. Stable LV-transduced NSC lines (at least 5 passages post-transduction) were used for transplantation experiments. Efficacy of NSC transduction was assessed on neurosphere bulk cultures by quantifying 1) the number of GFP-expressing cells by FACS; 2) the number of vector copies per genome (VCN) by qPCR; 3) GALC and ARSA enzymatic activity. The same analysis was performed in WT cells as well as in non-transduced and in cells transduced with control vectors. The effect of transduction on NSC functional features (survival, proliferation and differentiation capacity) was evaluated as previously described [3, 4]. For transplant experiments we selected cell preparations with ≥ 80% of GFP^+^ cells.

**Immunofluorescence and immunohistochemistry**

For immunofluorescence analysis, coverslips and free-floating vibratome sections were incubated in blocking solution (10% NGS + 0.3% Triton X-100 in PBS) for 1h at room temperature (RT) and then incubated overnight at 4°C with primary antibody in blocking solution. After 3 washings, antibody staining was revealed using species-specific fluorophore-conjugated secondary antibodies diluted in PBS+1% NGS. Controls consisted of sections incubated without primary antibodies. Coverslips and tissue sections were counterstained with 4', 6-diamidino-2-phenylindole (DAPI, Roche) or ToPro-3 (T3605, Invitrogen), washed in PBS, collected and mounted on glass slides using Fluorsave (CALBIOCHEM).

For immunohistochemistry, free-floating vibratome sections were incubated for 10 min in 3% H_2_O_2_ in methanol (only for lectins) or PBS. After 3 washing in PBS, sections were incubated in blocking solution (30 min) and then with appropriate primary antibodies or, for lectin histochemistry, in avidin (15 min) and biotin solution (15 min). These slices were subsequently incubated for 30 min with blocking solution (PBS+0.3%Triton-X+10% NGS) and then with Biotynilated Ricinus Communis Agglutinin I (RCA I, B-1085 Vector Laboratories; 1:200 in blocking solution) for 30 min.

After thorough washing with PBS and 5 min-incubation with 100 mM Tris-HCl staining was revealed using VECTASTAIN ABC kit (PK-6100Vector Laboratories). After washings in 100 mM Tris-HCl, the substrate 3-3 diamino-benzidine tetrahydrochloride (DAB, 167µg/ml in Tris-HCl 100mM + 3% H_2_O_2_) was added. Sections were monitored under the microscope for the appearance of precipitate. Reaction was stopped at the first appearance of brown colour in control sections (no primary antibody). Tissue sections were collected, mounted on glass slides and, after Cresyl Violet counterstaining, dehydrated and mounted with EUKITT.

**Kluver-Barrera staining**

Vibratome-cut slices were washed 3 times in PBS (5 min each), and incubated in 0.1% Luxol Fast Blue Solution (1g in 1L of 96% EtOH, 5 ml of 10% Glacial Acetic Acid) at 56°C overnight. Excess staining was rinsed off using 96% EtOH and then distilled water. Differentiation of white matter from the background was performed in 0.1% Lithium Carbonate Solution for 10-15 min. and continued in 70% EtOH until the grey matter was clear and white matter sharply defined (in blue). When differentiation was complete, slices were dehydrated and mounted with EUKITT.

**Cell counts and image acquisition**

The number of engrafted cells was assessed in 40µm-thick coronal brain sections (15-18 sections/mice, corresponding to one out of six series; 3-12 mice/treatment group) using anti-GFP and expressed as number of cells/section. This number was multiplied by six, in order to have an estimate of the total number of engrafted cells/brain. The percentage of engraftment was expressed as:  *(total number of engrafted cells / total number of transplanted cells) x100.*

The cell type composition of the transduced and/or cross-corrected cells in Twi recipients was assessed in coronal brain sections (2-4 slices/mice; *n=*3-5 mice/treatment group) selected within the region containing transduced cells using anti-GFP and anti-GALC antibodies in combination with lineage-specific markers and nuclear counterstaining in immunofluorescence followed by confocal microscopy analysis.

Z-stacks were recorded utilizing a PerkinElmer UltraVIEW ERS Spinning Disk Confocal (PerkinElmer Life Sciences, Inc.) using a 63×/NA1.4 Planapochromat oil-immersion lens (Carl Zeiss, Jena, Germany) with a 405 nm diode laser, 488 nm argon laser and a 568 nm krypton laser excitation wavelengths. Sequential confocal images were collected at 0.25 μm intervals covering 14 μm depth (56 total scanning images for each channel). The 3D signals and the orthogonal projection representations were obtained by means of Volocity Software (v5.2.1; PerkinElmer-Improvision, Lexington, MA, USA). Images were imported into Adobe Photoshop CS3 (USA) and adjusted for brightness and contrast.

3D reconstruction of NSC-injected brains was performed using a Leica DM 4000B microscope attached to a Neurolucida computer-assisted tracing system (Microbrightfield, Inc., USA). Images were imported into Adobe Photoshop CS3 or Image J software and adjusted for brightness and contrast.

For quantification of lectin histochemistry, CD68, Iba1 and GFAP immunohistochemistry, pictures of defined brain areas (see Supplementary Fig. 6) were sequentially acquired at 10X magnification using a threshold defined on the signal level of WT tissues (for lectins) or of tissues in which the primary antibodies were omitted (CD68, Iba1 and GFAP). Images were analyzed by the ImageJ software to quantify signal intensity corresponding to immunopositive areas (expressed as pixels), as previously described [5]. In order to minimize the bias and variability of the analysis, slices to be compared for signal quantification of a given marker were photographed and analyzed simultaneously.

**Syber Green qPCR**

Total RNA was isolated from tissues using TRIzol reagent (Invitrogen)/chloroform method and purified by RNeasy Mini Kit (Qiagen, Hilden, Germany). The quantity of RNA was determined by 260/280 nm optical density (OD) reading on a NanoDrop ND-1000 Spectrophotometer (NanoDrop, Pero, Italy). Reverse transcription was carried out using 1 μg of total RNA in the presence of 200 U of Quantitect Reverse Transcriptase kit (Qiagen, Hilden, Germany). Quantitative PCR analyses were performed with QuantiFast SYBR Green PCR Kit and QuantiTect Primer Assays (Qiagen) according to the manufacturer's protocol. PCR were run using an ABI7900HT apparatus (Applied Biosystems). The SDS 2.2.1 software was used to extract raw data. The difference between the threshold cycle (CT) of each gene and that of the endogenous controls β-Actin (ΔCT) was used to determine gene expression. To calculate the fold-change of gene expression between treated or untreated animals, we used an implemented analysis of covariance model (ANCOVA) as previously described [6, 7].

**qPCR for the detection of LV genome**

Genomic DNA was extracted from cells (Maxwell 16 Cell DNA Purification Kit) and quantified at NanoDrop ND-1000 Spectrophotometer (Euroclone, Pero, Italy). Vector copies per genome were quantified by TaqMan analysis starting from 100 ng of template DNA extracted from cells. Quantitative PCR was performed by amplifying the PSI sequence of the LV backbone using primers as follows: forward, 5’-TGAAAGCGAAAGGGAAACCA-3’, and reverse, 5’-CCGTGCGCGCTTCAG-3’. PCR product length was 64 bp at a final concentration of 750 nmol for forward and 200 nmol for reverse primers. The probe was 5’-VIC-AGCTCTCTCGACGCAGGACTCGGC- MGB-3’ at a 200 nmol final concentration. As internal reference for normalization, we amplified a fragment of the murine β-actin gene using the following set of primers and probe: forward primer,

5’-AGAGGGAAATCGTGCGTGAC-3’ at 300 nmol final concentration, reverse primer, 5’-CAATAGTGATGACCTGGCCGT-3’ at 750 nmol final concentration; the probe was 5’-VIC-CACTGCCGCATCCTCTTCCTCCCMGB- 3’ at 200 nmol final concentration. A standard curve of genomic DNA carrying 5 LV copies, validated by Southern blot analysis, was constructed using DNA extracted from transgenic mouse tissue. The standard curve, based on different dilutions of DNA (from 200 to 25 ng), and accordingly, of LV copies, was used as standard both for LV and for β-actin amplification. Reactions were carried out in a total volume of 25 μl, in an ABI Prism 7700 HT Sequence Detection System (Applied Biosystems). The VCN was calculated as follows: (ng LV/ng endogenous DNA) × (number of LV integrations in the standard curve).

**Brain and cell extracts**

Tissues were homogenized with an Elveheim type homogenizer in 10 mmol/L sodium phosphate buffer pH 6.0 with 0.1% (vol/vol) Nonidet NP40 and then subjected to 3 rounds of sonication. After 1 h, brain lysates were centrifuged (12.000g) in Eppendorf microfuges for 20 min. We used supernatants as tissue extracts for biochemical analyses. All procedures were carried out at 4 °C. Cells were harvested, washed in PBS, lysed for 1 h in 10 mmol/L sodium phosphate buffer, pH 6.0, containing 0.1% (vol/vol) Nonidet NP-40, and subjected to sonication. These steps were performed at 4°C. We measured protein content using the Bradford Protein Assay kit with bovine serum albumin as the reference standard.

**Supplementary Table 1. List of primary and secondary antibodies used.**

**Supplementary Table 2. Experimental groups of animals used for the different assays.**

The table shows the number of animals used in our study, splitted by genotype (WT and Twi mice) and by treatment:

- UT: untreated mice

- CTRLtWT and CTRLtTwi: WT or Twi mNSC transduced with bdLV.CTRL

- GALCtWT and GALCtTwi: WT or Twi mNSC transduced with bdLV.GALC

- hNSC: human NSC transduced with bdLV.CTRL.

Tissues of mice processed for histology were used for the cell counts, for immunofluorescence analysis and for histopathology. Tissues processed for molecular analysis were used for evaluation of GALC activity and for Q-PCR. Animals monitored for survival were also analyzed at PND40 for gait analysis and at the terminal stage for assessment of lectin storage. All the mice used for histology and molecular analysis were euthanized at PND40 with the exception of a small group (numbers in brackets) that were euthanized at PND7.

**References**

1. Amendola M, Venneri MA, Biffi A, et al. Coordinate dual-gene transgenesis by lentiviral vectors carrying synthetic bidirectional promoters. Nat Biotechnol*.* 2005;23:108-116.

2. Neri M, Maderna C, Ferrari D, et al. Robust generation of oligodendrocyte progenitors from human neural stem cells and engraftment in experimental demyelination models in mice. PLoS One*.* 2010;5:e10145.

3. Gritti A, Dal Molin M, Foroni C, et al. Effects of developmental age, brain region, and time in culture on long-term proliferation and multipotency of neural stem cell populations. J Comp Neurol*.* 2009;517:333-349.

4. Pluchino S, Gritti A, Blezer E, et al. Human neural stem cells ameliorate autoimmune encephalomyelitis in non-human primates. Ann Neurol*.* 2009;66:343-354.

5. Visigalli I, Moresco RM, Belloli S, et al. Monitoring disease evolution and treatment response in lysosomal disorders by the peripheral benzodiazepine receptor ligand PK11195. Neurobiol Dis*.* 2009;34:51-62.

6. Pucci F, Venneri MA, Biziato D, et al. A distinguishing gene signature shared by tumor-infiltrating Tie2-expressing monocytes, blood "resident" monocytes, and embryonic macrophages suggests common functions and developmental relationships. Blood*.* 2009;114:901-914.

7. Yuan JS, Reed A, Chen F, et al. Statistical analysis of real-time PCR data. BMC bioinformatics*.* 2006;7:85.
