# Supplementary material for: Neural Stem Cell Gene Therapy Ameliorates Pathology and Function in a Mouse Model of Globoid Cell Leukodystrophy
Source: Stem Cells. 2011 Aug 1;29(10):1559–71. doi: 10.1002/stem.701 (PMC3229988; doi:10.1002/stem.701)
Supplement: Supplementary file 10 [file stem0029-1559-SD10.doc]

| **PRIMARY ANTIBODIES** | | | |
| --- | --- | --- | --- |
| **Antigen** | **Host species, source and product number** | **Dilution** | |
|  |  | IF | IHC |
| CD68 | Rat IgG, Serotec MCA1957PE |  | 1:200 |
| Doublecortin (DCX) | Goat policlonal, Santa Cruz SC-8066 | 1:200 |  |
| Glial fibrillary acidic protein (GFAP) | Rabbit polyclonal, Dako ZO334  Mouse Monoclonal,Chemicon MAB3402 | 1:1,000  1:2,000 | 1:2,000 |
| Glutathione-S-transferase π (GST-π) | Rabbit poyclonal, MBL International 312 | 1:500 | 1:500 |
| Green Fluorescence Protein (GFP) | Rabbit polyclonal, Molecular probes A-11122  Chicken, Abcam AB-13970 | 1:500  1:1,000 | 1:1,000 |
| Iba-I | Rabbit polyclonal, Wako 019-19741 |  | 1:300 |
| Ki67 | Rabbit polyclonal, Novocastra NCL-KI67-P | 1:1,000 |  |
| Lysosomal-associated membrane protein 1 (LAMP1) | Rabbit polyclonal, Abcam AB24170 | 1:200 | 1:500 |
| Nestin | Mouse monoclonal IgG, Chemicon MAB353 | 1:500 |  |
| β-Tubulin IV | Mouse monoclonal IgG1 Sigma T7941 | 1:500 |  |
| Neuronal nuclei (NeuN) | Mouse monoclonal IgG1, Chemicon MAB377 | 1:250 |  |
| CNPase | Mouse monoclonal IgG1, Chemicon MAB326R | 1:1,000 |  |
| S100 β | Rabbit polyclonal, Swant 37A | 1:1,000 |  |
| Galactosylcerebrosidase  (GALC) | Chicken polyclonal, AP1021  Gift of Dr. C.W. Lee | 1:1,000 | 1:1,000 |
| Myelin Basic Protein  (MBP) | Rat monoclonal IgG, Chemicon MAB386 | 1:300 |  |
| Human influenza hemagglutinin epitope (HA) | Rat monoclonal IgG1, Roche 1867423001 | 1:50 |  |
| Human-specific nestin (hNestin) | Rabbit policlonal, Chemicon AB5922 | 1:500 |  |
| NG2 proteoglycan | Rabbit policlonal, Chemicon AB5320 | 1:300 |  |
| Adenomatous Polyposis coli (APC) | Mouse monoclonal IgG, Calbiochem OP80 | 1:500 |  |
| Oligodendrocyte transcription factor 2 (OLIG2) | Rabbit policlonal, Millipore AB9610 | 1:500 |  |
| Polysialylated-NCAM (PSA-NCAM) | Mouse monoclonal IgM, ABCYS, ABC0019 | 1:2,000 |  |
| **SECONDARY ANTIBODIES** | | | |
| **Conjugate** | **Source and product number** | **Dilution** | |
|  |  | IF | IHC |
| Alexa 488, Alexa 546,  Alexa 633 | Goat anti-mouse IgG A11001, A11003, A21050  Goat anti-rabbit A11008, A11010, A21070  Goat anti- chicken A11039, A11042  Molecular probes | 1:1,000  1:2,000  1:500 |  |
| Cy3 | Goat anti-mouse IgG 115-165-062  Goat anti-rabbit IgG 111-165-144  Jackson Lab. | 1:2,000  1:2,000 |  |
| Biotin | Goat anti-mouse 115-065-146  Goat anti-rabbit 111-065-003  Goat anti-rat 112-065-003  Goat anti-chicken 103-065-155 Jackson Lab. |  | 1:200  1:200  1:200  1:500 |

**Supplementary Table 1. List of primary and secondary antibodies used.**
